# Time-restricted eating versus calorie restriction for improving biomarkers of age in adults with overweight or obesity and incipient fatty liver disease: protocol for the ENSATI randomized controlled parallel groups trial

**DOI:** 10.3389/fendo.2026.1849550

**Published:** 2026-06-12

**Authors:** José A. Celada-Guerrero, Laura Rubio-Gordón, Yolanda Jiménez-Perez, Lorena López-Lora, Andrés López-González, Sara Delbuono, Ana Huertas, Diego Martínez-Urbistondo, José Ma Ordovás, Víctor de la O, Lidia Daimiel

**Affiliations:** 1 Nutritional Control of the Epigenome (NUCONEP), Precision Nutrition and Obesity Program, IMDEA Nutrition. CEI UAM + CSIC., Madrid, Spain; 2 CIBER de Fisiopatología de la Obesidad y Nutrición, Instituto de Salud Carlos III, Madrid, Spain; 3 Department of Endocrinology, Clínica Universidad de Navarra, Madrid, Spain; 4 Nutritional Genomics and Epigenomics Group, Precision Nutrition and Obesity Program, IMDEA Nutrition. CEI UAM + CSIC., Madrid, Spain; 5 Nutrition and Genomics Laboratory, Jean Mayer USDA Human Nutrition Research Center on Aging Tufts University, Boston, MA, United States; 6 Faculty of Health Sciences, International University of La Rioja (UNIR), Logroño, Spain; 7 Departamento de Ciencias, Farmacéuticas y de la Salud, Facultad de Farmacia, Universidad San Pablo-CEU, CEU Universities, Urbanización Montepríncipe, Boadilla del Monte, Spain

**Keywords:** biological age (BA), biomarkers of aging, calorie restriction (CR), epigenetic clock, fatty liver disease associated with metabolic dysfunction, geroscience, obesity, time-restricted eating

## Abstract

**Introduction:**

The increasing global lifespan has shifted the primary objective of geroscience from merely extending lifespan to maximizing health span. Biological aging is a gradual, time-dependent process marked by progressive cellular deterioration that culminates in increased vulnerability, frailty, morbidity, and mortality. Understanding the mechanisms that accelerate or decelerate this deterioration is crucial for developing effective interventions. Diet is recognized as the leading modifiable behavioral risk factor influencing the global burden of noncommunicable diseases and mortality. Therefore, nutritional interventions constitute a highly practical and scalable strategy for promoting healthy aging.

**Methods:**

The ENSATI trial is a randomized, open-label, controlled study with three parallel arms: active dietary counseling control, 25% calorie restriction, and time-restricted eating (14-hour fasting/10-hour eating window) over six months, followed by six months of post-intervention monitoring. A total of 177 adults aged 50–70 years with overweight/obesity and incipient fatty liver disease will be enrolled.

**Analyses:**

Primary outcomes include changes in body composition (dual X ray densitometry), hepatic fat (elastography) and metabolism (indirect calorimetry). Secondary outcomes encompass glucose regulation (continuous glucose monitoring), gut microbiome profiles, molecular biomarkers of aging (epigenetics, autophagy, immunosenescence), alongside psychological, cognitive, sleep, and dietary assessments using validated tools. Analyses will follow an intention-to-treat approach, with per-protocol sensitivity analyses and sex-stratified models. Mixed-effects models adjusted for potential confounders will assess intervention effects.

**Discussion:**

Current TRE and caloric restriction studies are limited by short durations, small samples, and poor control of energy intake, often lacking molecular biomarkers of aging. ENSATI overcomes these gaps through a 12-month, adequately powered, randomized, multi-arm design with rigorous dietary monitoring and comprehensive molecular and physiological profiling, enabling a more rigorous exploration of the relative contributions of caloric intake versus chronobiological effects on obesity and aging.

**Ethics and dissemination:**

This study was approved by IMDEA Ethics Committee (IMF PI-057). All participants will provide written informed consent. The findings will be disseminated in peer-reviewed scientific journals and at scientific conferences.

## 1 Introduction

Global life expectancy has doubled since 1900 and continues to rise. In 2020, the global population aged 60 years and over is just over 1 billion people, representing 13.5% of the world’s population. That number is 2.5 times greater than the 1980 estimates (382 million) and is projected to reach nearly 2.1 billion by 2050. But adding more years to life can be a mixed blessing if it is not accompanied by adding more life to years (1). The increase in lifespan has been accompanied by an increase in the prevalence of chronic non-communicable diseases (cancer, heart disease, diabetes, and hypertension, among others). that are also primary contributors to healthcare costs. Among the risk factors for these diseases, those related to lifestyle, including smoking, lack of physical activity, alcohol consumption and unhealthy diets, have the greatest impact. This makes the development of strategies to implement lifestyle improvements an urgent priority for an aging population (2, 3).

As the global population continues to age, understanding the distinction between chronological age and biological age becomes essential. Chronological age (CA) is a fixed and objective measure, defined solely by the linear passage of time from an individual’s birth. In contrast, biological age (BA) reflects the progressive decline in physiological functions essential for survival and reproduction (4). Unlike CA, biological aging is a dynamic and highly individualized process, influenced by a complex interplay of intrinsic factors—including genetic, epigenetic, metabolic, and microbiome-related components—and extrinsic factors such as psychological stress, sleep quality and duration, physical activity, and dietary patterns (5–8).The rate of biological aging progression can be assessed through established biomarkers of aging (BoA), which reflect a network of deeply interconnected processes that collectively shape systemic aging phenotypes (5). These biomarkers are grounded in the conceptual framework of the twelve hallmarks of aging (6). Together, these hallmarks underpin the development of BoA capable of quantifying the decline in physiological resilience and functional reserve, thereby providing a more accurate estimation of BA progression than CA.

Systemic modulators profoundly affect BA. For instance, obesity and biological aging are increasingly recognized as interrelated processes that influence each other. Pathological weight gain acts as a primary driver of accelerated biological aging. This acceleration is characterized by the premature emergence of these molecular hallmarks of aging, such as telomere attrition, mitochondrial dysfunction, impaired nutrient sensing, and cellular senescence (7–10). Lifestyle interventions that lead to weight loss, such as energy-reduced diets and fasting, have been shown to significantly improve molecular markers of aging, specifically by slowing telomere attrition, improving immunosenescence and upregulating autophagy pathways (11–15).

The influence of nutrition extends beyond basic metabolic support; it actively regulates the rate of molecular damage accumulation captured by BoA. Healthy dietary patterns such as Mediterranean diet (MedDiet) or the Alternative Healthy Eating Index (AHEI), have been strongly associated with healthy aging (16). The MedDiet has been shown to positively impact molecular hallmarks of aging including telomere attrition, dysbiosis and cellular senescence (11, 13, 17, 18). Beyond the nutritional composition of the diet, calorie restriction and fasting are emerging strategies to promote health span. Calorie restriction has been shown to improve cardiovascular risk factors such as LDL cholesterol and blood pressure (15) and modestly slow the pace of aging, though effects on BA estimates were small (19). Intermittent fasting also shows promise for delaying biological aging: alternate-day fasting reduces body weight and fat mass, though long-term adherence remains challenging (20) while periodic fasting (5:2) enhances ketogenesis and microbiota diversity (21). However, existing human trials are limited by small sample sizes and short intervention periods, underscoring the need for larger, longer studies to clarify fasting’s impact on biological aging. Time-restricted eating (TRE) is another fasting-based dietary approach that has gained recent attention. In a TRE protocol, all daily food intake is confined to a specific time window—typically ranging from 4 to 12 hours—while fasting is maintained for the remaining hours of the day. A 12-week intervention using a self-selected 10-hour TRE window in patients with metabolic syndrome significantly improved cardiometabolic health (22). In contrast, a 12-week 16:8 TRE regimen produced only modest weight loss, not significantly different from controls, with no improvements in metabolic markers and a slight reduction in appendicular lean mass (23). Evidence suggests early TRE is more effective than midday TRE, improving insulin sensitivity, fasting glucose, body mass, adiposity, inflammation, and gut microbiota diversity (24). However, a recent study found that TRE, regardless of timing, is safe and feasible but offers no additional benefit over a Mediterranean diet for reducing visceral fat (25). These short-duration studies (≤ 12 weeks) underscore the need for longer trials comparing TRE with calorie restriction in the context of healthy aging.

The overall aim of the ENSATI randomized controlled trial is to investigate the effects of a long-term 14:10 TRE dietary intervention compared with 25% traditional calorie restriction and an active dietary counseling control group on parameters associated with healthy aging, including body composition, metabolism, circadian rhythms and sleep, cognition, quality of life and BoA (autophagy, dysbiosis, immunosenescence and epigenetic alterations).

## 2 Material and methods

### 2.1 Patient and public involvement

Patients and the public have played an active role in shaping the design and conduct of this trial. During the planning phase, feedback was gathered from potential participants through informal interviews and focus groups to ensure that the intervention was practical, acceptable, and aligned with their needs. The website also serves as a communication platform, providing updates, FAQs, and accessible summaries of progress and results. Throughout the study, participants will be encouraged to share their experiences and suggestions, which will inform ongoing improvements in trial procedures. At the reporting stage, a plain-language summary of the findings will be distributed to all participants and published on the website to ensure transparency and accessibility. Additionally, results will be shared with patient advocacy groups and healthcare professionals to promote knowledge transfer and practical application. This collaborative approach aims to enhance participant engagement, improve trial relevance, and maximize the impact of the research on public health.

### 2.2 Trial design

The ENSATI study is an exploratory randomized, open-label, controlled parallel groups trial with three intervention arms. The three intervention arms are: an active dietary counseling control group (CG), a traditional 25% calorie restriction group (CRG) and a moderate ‘10-hour Time-Restricted Eating (TRE)TRE group (TRE). The study has been prospectively registered in ClinicalTrials. Gov (NCT05880095). This protocol is reported following Standard Protocol Items: Recommendations for Interventional Trials (SPIRIT) guidelines (26) and results will be reported following the Consolidated Standards of Reporting Trials (CONSORT) guidelines (27).

### 2.3 Participants’ eligibility criteria

Eligible participants are men and women, 50–70 years old, with overweight or obesity (body mass index, BMI, 25–40 Kg/m^2^) and incipient liver disease as assessed by a fatty liver index (FLI index ≥ 30). Other inclusion criteria are habitual eating window ≥ 12 hours, stable sleeping rhythms and stable weight in the last 3 months prior to study inclusion. **Table 1** shows the detailed list of inclusion and exclusion criteria.

**Table 1:** Inclusion and exclusion criteria of the ENSATI trial.

Inclusion criteria	Exclusion criteria
Men and women	Failure to meet inclusion criteria
Age: 50–70 years	Women of reproductive age without established menopause (no menstrual period in previous 12 months)
BMI: 25–40 kg/m²	Excessive alcohol consumption (CAGE questionnaire score >2; Ewing, 1984; Malet et al., 2005)
Incipient fatty liver disease detected by ultrasound or biomarkers (Fatty Liver Index score ≥30 if ultrasound screening is unavailable). *Reference: EASL Clinical Practice Guidelines for the management of non-alcoholic fatty liver disease, 2016.*	Smokers who changed smoking habits in the 6 months prior to study start (including starting or quitting)
Habitual daily eating period at baseline ≥12 hours	Diagnosed kidney disease
Regular sleep cycle in the month prior to study start (7 ± 2 hours per day)	Prevalent cardiovascular disease or angina
Stable weight in the 3 months prior to study start (weight change ≤4 kg)	Liver disease other than non-alcoholic fatty liver disease
No plans to change physical activity level in the 6 months following study start	Uncontrolled endocrine disorders (hypothyroidism, hyperthyroidism, type 1 or type 2 diabetes, adrenal gland disease)
Not following a weight-loss diet	Uncontrolled hypertension
	Pancreatitis
	Under medical treatment affecting weight, food intake, energy expenditure, or sleep in the 3 months prior to study start
	Food allergies or intolerances preventing adherence to the intervention protocol
	Diagnosed eating behavior disorders
	Shift workers
	Participation in another study that may interfere with the current study
	Social, cultural, or psychological factors that may affect adherence to the intervention protocol (e.g., inability to consume solid foods, non-permanent residence, institutionalization)

**Figure 1:**
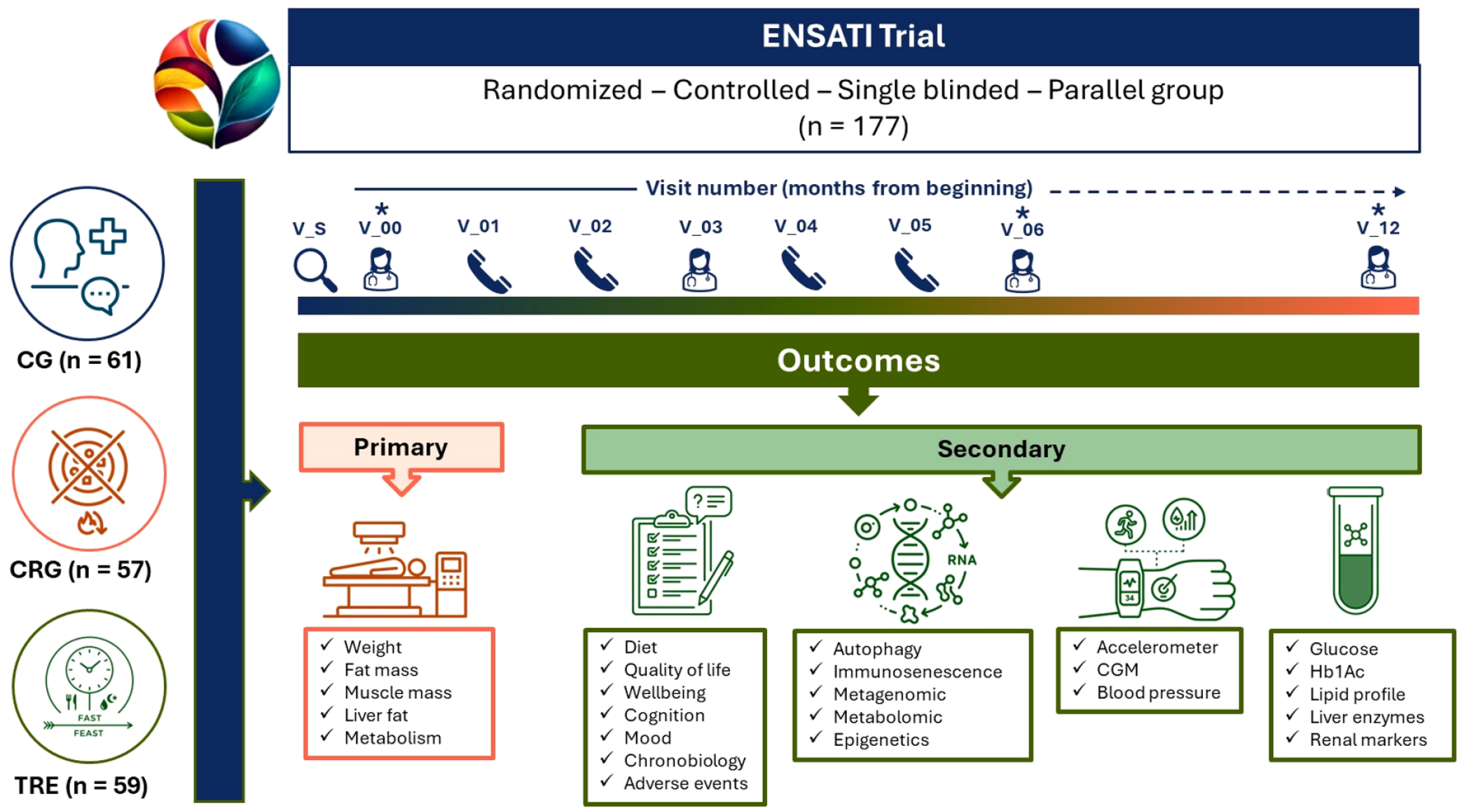
ENSATI trial design V_S refers to the screening visit, and V_00 denotes the baseline visit (1-week after V_S). Subsequent visits are numbered according to the month of follow-up from baseline. The dashed line represents the 6-month period without intervention. CG = Active dietary counseling Control Group; CRG = Caloric Restriction Group; TRE = Time-Restricted Eating Group. Sample size for each intervention arm is shown in brackets. Asterisks indicate visits where the full evaluation protocol is applied. The adverse events questionnaire is administered at every visit, regardless of format (in-person or telephone). Similarly, the MEDAS questionnaire and meal-time records are collected at all study visits. * Some icons in the image have been produced with generative AI tools.

Participant eligibility will be assessed during the screening visit (**Figure 1**). This visit will include a physical examination with anthropometric measurements to confirm BMI and FLI thresholds, an interview to evaluate the stability of sleep and eating habits, the habitual eating window (≥12 hours), and the participant’s commitment and ability to follow the dietary protocols. Medical records, including biochemical parameters, will be reviewed to verify FLI criteria and identify any medical conditions or current medications.

The trial is community-based and conducted in Madrid, Spain. Participants were recruited from patients referred by collaborating general practitioners and nurses from primary healthcare centers. Recruitment took place across four primary healthcare centers located in Madrid following approval from the Central Research Committee of the Health Council. General practitioners and nurses at these centers conduct pre-screening by reviewing medical records. Eligible individuals were then contacted by phone to explain the study and invite participants. Those who agree were scheduled for a screening visit to confirm eligibility. All study visits, including baseline assessments, follow-up visits, and intervention delivery, were conducted in IMDEA Nutrition facilities. Biological samples and blood analyses were collected in these community healthcare settings to ensure accessibility and integration with routine care.

During the screening visit, participants received comprehensive oral and written information about the study and were given sufficient time to review the materials and ask questions. Informed consent was then obtained prior to any study procedures. For those who consent, the visit proceeded with a physical examination, including blood pressure, height, weight, and body composition assessed by bioelectrical impedance. Medical records were reviewed, and an interview was conducted to verify compliance with inclusion and exclusion criteria, as well as the participant’s ability and commitment to follow the dietary protocols.

### 2.4 Interventions

#### 2.4.1 Control group/active dietary counseling control group

Participants randomized to the active dietary counseling control group (CG) will be instructed to maintain their usual their usual eating schedule with no mandated calorie or time restrictions. However, given their condition of being overweight or obesity, and to comply with ethical standards established by the Ethics Committee for this kind of population, they will receive general recommendations to follow a healthy Mediterranean-style dietary pattern. Therefore, this arm serves as an active dietary counseling control rather than a passive ‘usual care’ group. Following the same visit schedule as the intervention arms to ensure comparable intensity of contact, participants in the CG will attend individual nutritional education sessions of approximately 20 minutes throughout the study to reinforce adherence to the Mediterranean diet. Nutritional counseling will focus on three key aspects: (a) understanding the principles of the Mediterranean dietary pattern, (b) learning to interpret food labels, and (c) developing skills to plan meals that align with a Mediterranean diet.

#### 2.4.2 Calorie restriction group

Before the intervention begins, total energy expenditure will be estimated for all participants based on resting metabolic rate and physical activity level. A Mediterranean diet plan with a 25% energy reduction will then be individually designed and implemented for participants allocated to the Caloric Restriction Group (CRG). Instructions for the CRG will emphasize daily adherence to the prescribed plan, and participants will receive weekly meal plans, recipes, and shopping lists to facilitate compliance. Nutritional counseling will encourage participants to improve their score on the Mediterranean Diet Adherence Screener (MEDAS) (28), with particular attention to items initially scored as “0.”

#### 2.4.3 Time-restricted eating group

Participants allocated in the TRE group will follow a Mediterranean-style diet without energy restriction during a designated 10-hour eating window, leaving a 14-hour fasting period. Each participant may choose the 10-hour eating window that best fits their habits, within the recommended range of 06:00 am to 08:00 pm. This 10-hour window is operationalized to achieve a significant advance in meal timing within the Spanish context. Due to Spain’s geographical location in the Central European Timezone (CET)—which is one hour ahead of its solar time—and cultural habits, peak dinner times typically occur between 21:00 and 20:30. Consequently, a 20:00 cutoff requires participants to advance their last meal by approximately two hours, effectively reducing nocturnal eating and improving alignment with the solar cycle. This approach balances physiological rigor with the feasibility and long-term sustainability required for a 6-month intervention in a free-living Mediterranean cohort. During fasting periods, only water and other non-caloric beverages will be allowed. The time-restricted eating schedule must be maintained consistently on both weekdays and weekends.

### 2.5 Criteria for discontinuation or modification

Allocated interventions may be discontinued or modified under specific circumstances, including participant request, relocation that prevents attendance at study visits, occurrence of adverse events, initiation of weight-loss medication, or significant changes in health status that compromise safety or adherence. Adjustments may also be made if clinical assessments indicate worsening or improvement of the participant’s condition that require a different approach. All decisions will be documented and communicated to the participant, and, when appropriate, discussed with the study’s clinical team to ensure ethical and medical compliance.

During the trial, participants may continue receiving routine medical care and standard treatments for conditions unrelated to the study intervention. However, initiation of weight-loss medications, participation in other dietary or lifestyle intervention programs, or use of supplements specifically intended for weight management will be prohibited, as these could interfere with study outcomes. Any changes in medication or health status will be documented and reviewed by the study team to ensure protocol compliance and participant safety.

### 2.6 Strategies to improve and monitor adherence

To promote adherence to the intervention protocols, participants will receive individualized nutritional counseling, weekly meal plans, recipes, and shopping lists. Behavioral strategies, such as goal setting and regular feedback, will be incorporated during follow-up visits. Adherence will be monitored through dietary diaries, where participants record all meals and mealtimes, and by completing the MEDAS at each follow-up visit. These tools will allow the research team to evaluate compliance with both the dietary pattern and the prescribed eating schedule, enabling timely reinforcement or corrective actions when needed.

An intensive follow-up schedule has been designed to promote adherence, detect protocol deviations early, and implement corrective actions (**Figure 1**). At the screening visit (V_S), recruited participants will be instructed to maintain their usual dietary and physical activity habits until the baseline visit. Then, participants will attend a baseline visit (V_00) within one week, where inclusion/exclusion criteria will be reconfirmed, group allocation communicated, and intervention materials provided with detailed adherence instructions. At this visit, comprehensive assessments will be performed, including anthropometry, body composition, hepatic elastography, indirect calorimetry, biological sample collection, and blood analysis. Participants will be asked to record the timing of all meals throughout the intervention and to complete three-day dietary records—covering two weekdays and one weekend day—at baseline, midterm (3 months, V_03), and at the end of the intervention (6 months). At the start of the study, participants will receive detailed instructions on how to accurately report dietary intake, supported by educational materials that include examples and portion size guides. These records will be used to calculate total energy intake and macronutrient composition, including carbohydrates, fats, and proteins. This approach will enable us to assess the degree of energy restriction in the CRG and to evaluate the eating window in the TRE group.

Monthly follow-up contacts will be scheduled. At months 1 (V_01), 2 (V_02), 4 (V_04), and 5 (V_05), follow-up will consist of telephone calls to monitor adverse events, identify barriers, and apply corrective measures if needed. At month 3, an in-person visit will assess anthropometry, lifestyle data, adverse events, and protocol compliance.

The intervention lasts six months. The final intervention visit will include full assessments: anthropometry, body composition, hepatic elastography, indirect calorimetry, blood analysis, and biological sample collection. A post-intervention visit at 12 months (V_12, six months after intervention completion) will evaluate long-term adherence and effects without directed counseling, using the same protocol as baseline and 6-month visits. Accelerometers and continuous glucose monitoring devices will be applied at baseline, 6 months, and 12 months.

### 2.7 Outcomes

All measurements will be performed by a dedicated team of trained staff to ensure consistency and reliability. In addition to baseline and final assessments, anthropometry and body composition (via bioelectrical impedance) will also be measured at month 3, the mid-point of the intervention (**Figure 1**). These intermediate evaluations will allow us to capture changes over time and assess the trajectory of outcomes throughout the six-month intervention period. A comprehensive overview of all study outcomes and variables is provided in **Table 2**.

**Table 2:** ENSATI participant's timeline: Schedule of enrollment, interventions and assessments.

	Trial period								
Timepoint	Enrollment		Post-randomization (months)						
	S	Baseline	1M	2M	3M	4M	5M	6M	12M
Enrollment									
Informant consent	1								
Eligibility questionnaire	1								
Randomization		1							
General questionnaire		1			2			3	4
Intervention									
CG	1	2	3	4	5	6	7	8	9
CRG	1	2	3	4	5	6	7	8	9
TRE	1	2	3	4	5	6	7	8	9
Assessments									
Anthropometric measurements^1^	1	2			3			4	5
Bioelectrical impedance	1	2			3			4	5
Dual X-Ray densitometry (DXA)		1						2	3
Hepatic elastrography^2^		1						2	3
Indirect calorimetry^3^		1						2	3
3-Day food register		1			2			3	4
Food frequency questionnaire^4^		1							2
Mediterranean diet questionnaire (17-items)^5^		1	2	3	4	5	6	7	8
Physical activity questionnaire^6^		1			2			3	4
Accelerometers (activity and sleep)^7^		1						2	3
Health Survey^8^		1			2			3	4
Wellbeing^9^		1			2			3	4
Psychopathological questionnaires^10^		1			2			3	4
Cognitive questionnaires^11^		1						2	3
Blood sample collection		1						2	3
Blood pressure measurement	1	2			3			4	5
Glucose records^12^		1						1	1
Adverse effects			1	2	3	4	5	6	
Biological samples collection^13^		1						2	3

M, month, S, selection. 1 Anthropometric measurement include: weight, height, waist circumference and hip circumference; 2 FibroScan Mini 430; 3 MetaLyzer 3B; 4 136-item from PREDIMED-Plus Trial; 5 17-item energy-restricted Mediterranean diet questionnaires from PREDIMED-Plus Trial; 6 IPAQ and the REGICOR Short Physical Activity questionnaire; 7 FitBit versa 4; 8 SF-36 quality of life scale; 9 W-BQ12; 10 Beck Depression Inventory (BDI-II), multidimensional scale of weight locus of control and lifetime eating disorders diagnostic criteria; 11 STROOP and verbal Rey test; 12 FreeStyle Libre 3 Abbott; 12 Urine.

The study’s primary outcome are changes from baseline to 6 months in weight, body composition (fat mass, muscle mass, lean mass), hepatic fat and metabolism. Secondary outcomes include changes from baseline to 6 months in cardiometabolic markers (i.e., glucose, hemoglobin A1c, lipid profile, liver and kidney function, markers and estimated glomerular filtration rate), 24-hour glucose curves (via CGM), fecal microbiota, sleep and physical activity patterns, psychological outcomes (i.e., depression, stress, anxiety, mood, chronodisruption and wellbeing), cognition, self-reported health status, dietary habits and eating behavior assessment. Other secondary outcomes include molecular biomarkers of aging (BoA) such as epigenetics, autophagy and immunosenescence.

#### 2.7.1 Primary outcomes

##### 2.7.1.1 Anthropometry and body composition

Anthropometric and body composition measurements include height, weight, waist circumference, and segmental body composition (upper limbs, lower limbs, and trunk). Height will be measured using a wall-mounted stadiometer. Weight and body composition will be assessed by electrical bioimpedance (BIA) with a Tanita RD545 device. Waist circumference will be measured at the midpoint between the last rib and the iliac crest after a deep exhalation, using a flexible measuring tape. Anthropometric measurements will be taken with participants barefoot and wearing light clothing.

Additionally, body composition will be assessed by dual-energy X-ray absorptiometry (DXA) using a Hologic W device at visits V_00, V_06, and V_12. A whole-body scan will be performed, and body composition will be analyzed across seven segments: head, trunk, right arm, left arm, right leg, left leg, and the android/gynoid region. DXA measurements will be conducted by personnel certified to operate radiological equipment.

##### 2.7.1.2 Liver fat content

The effect of the intervention on liver health will be assessed using hepatic elastography performed with FibroScan^®^ equipped with SmartExam and Controlled Attenuation Parameter (CAP) technology to determine the degree of hepatic steatosis and fibrosis. FibroScan^®^ is a non-invasive diagnostic tool that uses transient elastography to measure liver stiffness, which correlates with the degree of fibrosis, and CAP to quantify fat accumulation in the liver. This method is widely validated as an alternative to liver biopsy for staging fibrosis and detecting steatosis, offering high reproducibility and patient safety (29). Measurements will be performed by trained personnel following standardized procedures to ensure accuracy. Liver stiffness will be expressed in kilopascals (kPa), and CAP values in decibels per meter (dB/m), providing quantitative data for evaluating the impact of dietary interventions on hepatic health.

##### 2.7.1.3 Energy expenditure and metabolism

Energy expenditure will be estimated using the Harris-Benedict equation and objectively measured through resting indirect calorimetry with the Metalyzer system. A 30-minute gas exchange assessment will be performed, during which oxygen consumption (VO_2_) and carbon dioxide production (VCO_2_) will be continuously recorded to calculate resting metabolic rate (RMR). The Cortex software will automatically select the 5-minute interval that provides the highest data quality, ensuring a quality score above 40 out of 100, in accordance with device and software specifications. Indirect calorimetry is considered the gold standard for measuring energy expenditure, as it provides precise metabolic data by analyzing respiratory gases under controlled conditions.

#### 2.7.2 Secondary outcomes

##### 2.7.2.1 Exploratory biological profiling: biomarkers of aging

**i. Epigenetics assessment**

DNA will be extracted from PBMCs using the QIAamp Blood Mini Kit (Qiagen, Venlo, Netherlands) according to the manufacturer’s instructions. DNA methylation profile will be studied with the Infinium Methylation EPIC BeadChip v.0 (Illumina San Diego, CA, USA),

Preprocessing DNAm data includes background correction, quantile normalization, and probe bias correction. The beta methylation values will be calculated using the Regression on Correlated Probes (RCP) method (30). The estimated beta values will then be converted to M-values. Quality control procedures will include the removal of probes with > 5% missing data and samples with >1% missing data as well as filtering out probes targeting SNP-containing sites, cross-reactive probes, and probes mapping to sex chromosomes unless required for specific analyses. Signal intensities, bisulphite conversion efficiency, and array-specific control probes will be manually inspected. Epigenetic age will be estimated using first-, second- and third-generation DNA methylation clocks, including Horvath’s clock (31), GrimAge (32), and PhenoAge (33) as well as Dunedin PACE (34). In addition, telomere length will be inferred from DNA methylation data using established computational algorithms (35).

Downstream bioinformatic analyses will include identification of differentially methylated positions (DMPs) and differentially methylated regions (DMRs) as well as pathway and gene-set enrichment analyses (e.g., GO, KEGG, Reactome). Longitudinal analyses will be performed with mixed-effects models to evaluate temporal changes in DNAm. Integrative multi-omics analyses will incorporate microbiome, metabolomic, and clinical variables, using multivariate approaches and machine-learning algorithms to explore mechanistic links between methylation patterns, metabolic outcomes, and intervention adherence.

**ii. Immunosenescence**

To assess immunosenescence, the percentage of CD28- T lymphocytes will be measured by flow cytometry. Immunostaining and immunosenescence will be performed in previously isolated PBMCs. To account for technical variability, a control sample without staining containing a pool of different plasma samples will be used in each measurement, as well as an FMO (fluorescence minus one controls) sample for CD3 and CD28. Samples will be analyzed with a FACSCelesta SORP cytometer (BD Bioscience, San Jose, California, USA) and with BD FACSDiva v9.0 software. Analysis of data obtained from the cytometer will be performed with the FlowJo v10.6.2 (BD Bioscience San Jose, California, USA).

**iii. Measurement of autophagy by flow cytometry and fluorescence microplate reader**

The ENSATI trial evaluates autophagic activity in peripheral blood mononuclear cells using the CYTO-ID^®^ Autophagy Detection Kit 2.0 (Enzo Life Sciences, Lausen, Switzerland), with a focus on measuring true autophagic flux rather than simple autophagosome accumulation. PBMCs serve as valuable organotypic sensors because they reflect an individual’s unique nutritional, endocrine, and genetic environment. Unlike invasive tissue biopsies, PBMCs are routinely isolated from whole blood, allowing for repeatable longitudinal assessments. They are sensitive to systemic aging hallmarks, metabolic conditions, and lifestyle changes. When optimized with procedural safeguards and lysosomal inhibitors like chloroquine, PBMC-based assays provide a methodologically robust, high-resolution platform to investigate dynamic autophagic flux. This offers a critical “biological window” into how nutritional interventions modulate cellular recycling pathways.

The assessment of the autophagic flux is achieved through ex vivo treatment with chloroquine (60µM), which blocks lysosomal fusion and allows comparison of accumulated autophagic signal against vehicle-treated baseline samples. Rapamycin (500 nM) is included as a positive control to induce autophagy and confirm cellular responsiveness. After treatment, cells will be washed and incubated with Microplate Dual Detection Reagent (CYTO-ID^®^ Green and Hoechst 33342) for 30 minutes at 37 °C, followed by washing and processing by flow cytometry or fluorescence microplate reader. To ensure robustness and reproducibility of this biomarker, careful procedural controls are applied, including serum supplementation to protect PBMCs during handling, precise normalization of cell density to ensure fluorescence reflects autophagic vesicle content per cell, and exclusion of cytotoxic conditions that could compromise biological validity. Fluorescence measurements are performed in triplicate using a microplate reader and validated by flow cytometry. Together, this approach enables sensitive detection of autophagic flux and supports investigation of whether dietary interventions such as time-restricted eating or caloric restriction activate distinct cellular recycling pathways independently of weight loss.

For fluorescence microplate analysis, treated and labeled PBMCs cells will be seeded in black 96-well plates and read on a microplate reader using FITC and DAPI filter sets. All measurements will be performed in duplicate, and samples will be randomly assigned to wells to minimize positional bias. Each plate will include blank wells, negative controls, and positive controls to ensure assay validity.

For flow cytometry, samples will be analyzed with a FACSCelesta SORP cytometer (BD Bioscience, San Jose, California, USA) and with BD FACSDiva v9.0 software. Analysis of data obtained will be performed with the FlowJo v10.6.2 (BD Bioscience San Jose, California, USA).

##### 2.7.2.2 Cardiometabolic risk markers

Venous blood samples will be collected and processed to separate plasma from blood cells. Samples will be stored at −80 °C to preserve integrity for subsequent analyses. Peripheral blood mononuclear cells (PBMCs) will be isolated using Lymphoprep™ (STEMCELL Technologies, Vancouver, Canada) and cryopreserved for future research. A comprehensive panel of cardiometabolic markers will be assessed, including fasting glucose, hemoglobin A1c, and lipid profile (total cholesterol, HDL cholesterol, triglycerides). LDL cholesterol will be calculated using the Friedewald formula. Liver and kidney function markers (alanine aminotransferase, gamma-glutamyl transferase, bilirubin, creatinine) and estimated glomerular filtration rate will also be measured, along with a complete blood count. Analyses will be performed either at participating primary healthcare centers or outsourced to certified laboratories. Additionally, systolic and diastolic blood pressure will be measured using an automated monitor (M2, Omron Healthcare Europe B.V., Hoofddorp, Netherlands) following the 2021 European Society of Hypertension guidelines, with three readings taken after 5 minutes of rest (36).

##### 2.7.2.3 Glycemic control

We aim to evaluate the impact of the interventions on dynamic glycemic control using CG). Participants will wear a CGM device (FreeStyle Libre 3, Abbott Laboratories, Abbott Park, IL) for two weeks at three time points: baseline (7 days before and 7 days after starting the intervention), post-intervention (month 6), and post-follow-up (month 12). CGM data will be analyzed to calculate key glycemic control metrics, such as 24-hour mean glucose, following the latest international consensus guidelines (37). Additionally, advanced glucose variability metrics will be derived to characterize the size and shape of postprandial glucose curves and the dynamics of glucose fluctuations, providing a comprehensive assessment of glycemic patterns beyond traditional measures.

##### 2.7.2.4 Fecal microbiota

To comprehensively assess fecal microbiome diversity, composition, and functional potential, stool samples will be collected at baseline, 6 months, and 12 months. Microbial DNA will be extracted using the Qiamp Fast DNA Stool Kit (Qiagen, Venlo, Netherlands) following manufacturer’s instructions, and quality will be assessed via Nanodrop 2000. Shotgun metagenomic sequencing will be performed on Illumina platforms, targeting ≥10 million paired end reads per sample. Bioinformatic processing includes quality filtering, host DNA removal, and taxonomic profiling. Diversity metrics (alpha and beta) will be computed, and functional annotation will be conducted. Statistical analyses will assess longitudinal changes using linear mixed-effects models and differential abundance analyses. Integration with clinical data will employ multivariate methods and machine learning approaches to identify associations between microbiome features, dietary adherence, and metabolic outcomes. Quality assurance includes technical replicates, negative controls, and batch effect correction. Optional extensions include metabolomics, metatranscriptomics, and strain-level analyses to provide deeper insights into microbial function, host–microbiome interactions, and ecological dynamics.

##### 2.7.2.5 Circadian rhythms and sleep

To evaluate the impact of the intervention on circadian rhythm and sleep quality, we will employ a multimodal approach combining validated questionnaires and objective measurements. Subjective sleep quality will be assessed using the Pittsburgh Sleep Quality Index (PSQI), which provides a global score based on seven components of sleep (38), and the Morningness-eveningness questionnaire (MEQ) to determine chronotype (39) In addition, objective sleep parameters will be monitored using accelerometric data collected by the Fitbit Versa 3 (Google, Dublin, Ireland) (40) to calculate the average sleep timing and sleep efficiency. Participants will wear the device continuously for 14 days at baseline and at 6 and 12 months of follow-up to record sleep timing, duration, and efficiency. Data will be extracted through the Fitbit platform and processed to calculate average sleep onset, wake time, and sleep efficiency across the monitoring period.

##### 2.7.2.6 Cognition, health status, mood and wellbeing

To comprehensively evaluate the psychological and cognitive effects of the intervention, participants will complete a battery of validated instruments administered at baseline and at 6 and 12 months of follow-up. Anxiety symptoms will be assessed using the Hamilton Anxiety Rating Scale (HARS) (41), a clinician-administered questionnaire requiring approximately 10 minutes. Emotional eating behaviors will be measured through the Emotional Eater Questionnaire (EEQ) (42), a self-reported tool taking about 10 minutes. Mood states will be evaluated using the Scale for Mood Assessment (EVEA) (43), a brief self-report instrument requiring approximately 2 minutes. Health status and function will be assessed with the SF-36 questionnaire (44), which captures eight dimensions of physical and mental health and takes about 10 minutes to complete. Additionally, overall wellbeing will be measured using the WQ-12 Wellbeing Questionnaire (45, 46), a concise self-report instrument designed to assess psychological wellbeing in approximately 5 minutes.

Cognitive performance will be examined using two standardized neuropsychological tests: the Rey Auditory Verbal Learning Test (RAVLT) (47), which evaluates verbal learning and memory and requires 10–30 minutes, and the Stroop Color and Word Test (SCWT) (48), assessing selective attention and cognitive flexibility, with an estimated duration of 10 minutes. Tests requiring administration by a trained researcher (HARS, RAVLT, SCWT) will be conducted in a controlled environment, while participants will individually complete self-reported questionnaires. This multimodal approach ensures a robust assessment of emotional, behavioral, cognitive, and wellbeing domains relevant to the study objectives.

### 2.8 Dietary data and other cofounding factors

Because unintentional reductions in energy intake of approximately 10%–30% (equivalent to ~300–500 kcal/day) have been reported when participants restrict their eating window to 4–10 hours per day (49), we will closely monitor and analyze changes in energy intake throughout the intervention period. Participants will be asked to record the timing of all meals throughout the intervention and to complete three-day dietary records—covering two weekdays and one weekend day—at baseline, midterm (3 months), and at the end of the intervention (6 months). At the start of the study, participants will receive detailed instructions on how to accurately report dietary intake, supported by educational materials that include examples and portion size guides. In addition, potential confounding factors such as educational level, employment status, smoking habits, and medication use will be assessed during the baseline visit through a structured interview, and any changes in these variables will be monitored throughout the intervention period.

### 2.9 Adverse events

Adverse events will be systematically assessed throughout the intervention using the ENSATI Adverse Events Questionnaire, designed to identify potential side effects related to the study. The questionnaire includes a checklist of common symptoms (e.g., fatigue, headaches, mood changes, gastrointestinal disturbances, hypoglycemia) and open fields for reporting other events or food intolerances. It also records whether the event is considered related to the intervention. Data collection will occur at six predefined time points: months 1, 2, 4, and 5 via structured telephone interviews, and months 3 and 6 through in-person interviews conducted by trained research staff. Each interview will follow a standardized protocol to ensure consistency and minimize interviewer bias. Responses will be documented electronically immediately after each contact in a secure database. The estimated completion time for each questionnaire is approximately 4 minutes, reducing participant burden while ensuring comprehensive monitoring. Any serious or unexpected adverse events will be reported promptly to the principal investigator and the ethics committee according to regulatory requirements.

### 2.10 Sample size

The sample size calculation variable is the percentage of weight lost compared to baseline. We selected this variable based on previous studies consistently showing that participants undergoing a TRE intervention reduce their weight by approximately 3% (22, 50–53). Our clinical trial will follow an open-label, randomized, controlled, parallel-group design, using a two-way ANOVA test to compare changes in variables between the three intervention arms. Only the investigator responsible for analyzing the results will remain blind to participants’ intervention assignments. The trial will otherwise be open label for both participants and the investigators administering the intervention protocols. The sample size calculation assumes three intervention groups, a 5% difference in weight loss between groups, and a within-group variability of 50%. We set a significance level of 0.05 (Type I error) and a power of 80% (Type II error). The calculation was performed using the power.anova.test function in R version 4.1.3 (2022-03-10) via RStudio. Based on these parameters, 49 participants per group are required. Assuming a 20% dropout rate, we will need 59 participants per group to maintain statistical significance for the proposed parameter. Therefore, we will recruit 177 participants procuring a 1:1 sex ratio.

The choice of percentage weight loss as the primary powering variable is justified by its nature as a traceable trait that is both robust and directly linked to biological aging. Unlike molecular biomarkers, which are characterized by high inter-individual variability and are more difficult to track consistently during long-term follow-up in free-living populations, body weight provides a reliable clinical benchmark for intervention efficacy. Furthermore, because the ENSATI trial utilizes a comprehensive biological profiling approach—integrating a combination of measures including epigenetic clocks, autophagic flux, and immunosenescence—we decided not to focus the sample size calculation on a single molecular marker. This allows the trial to capture the multi-modal nature of biological aging while maintaining a single, coherent hierarchy centered on a validated clinical outcome. Additionally, there is current a lack of reference trials in similar populations whose sample size calculations are based on molecular BoA. However, our choice is supported by preliminary findings from a similar cohort (n=116) where significant improvements in immunosenescence and HDL functionality were detected with a smaller sample size (13). Thus, our target of 177 participants provides more than adequate power for molecular secondary outcomes.

While the study is formally powered for percentage weight loss, the resulting sample size of 177 participants is intended to provide a high-resolution platform for the investigative exploration of secondary molecular BoA. This robust cohort moves the evidence beyond standard pilot-scale geroscience trials, allowing for a more substantive exploration of whether nutritional interventions can modulate the rate of biological deterioration in high-risk populations.

### 2.11 Recruitment strategy

To ensure we reach the target sample size, we have implemented robust recruitment strategies based on close collaboration with general practitioners and nurses from primary care centers. Collaborating with primary care centers leverages the established patient–provider relationship, which is built on trust and continuity of care. General practitioners and nurses are familiar with patients’ medical histories and personal circumstances, allowing them to identify suitable candidates and address concerns effectively. This personalized approach fosters confidence in the study’s safety and relevance. Direct communication from trusted healthcare professionals also increases willingness to participate and strengthens commitment, as patients perceive the intervention as an extension of their routine care rather than an external research initiative. This strong confidence in the effectiveness of our recruitment strategy is grounded in our previous experience and proven success in similar studies (54).

### 2.12 Randomization and blinding

Participants will be randomly assigned to the different intervention arms using a complete block randomization method. The randomization blocks will be sex (male/female) and age range (50–59 years/60–70 years). This approach ensures that all groups will be comparable in terms of sex and age. Participants belonging to the same household will be identified and a cluster randomization approach will be applied to allocate household units to the same intervention arm. Participants will be randomly assigned to one of the three intervention arms (CG, CRG, or TRE) using a parallel-group design with an equal allocation ratio (1:1:1). Variable block sizes (4 to 16 participants) will be employed based on monthly recruitment rate to maintain allocation concealment and prevent predictability of group assignments. The randomization strategy has been designed to ensure allocation concealment and balance across groups. Randomization will be conducted using the randomizr package in R. No member of the research team administering the intervention and monitoring participants will have access to the randomization blocks or the randomizr script.

Due to the nature of the nutritional interventions (time-restricted eating and calorie restriction), the study adopts an open-label design for participants and staff directly involved in intervention delivery. Participants are informed of their assigned intervention arm at the baseline visit (V_00). Likewise, personnel responsible for nutritional counseling and for conducting anthropometric assessments, liver elastography, and indirect calorimetry are necessarily aware of group allocation. To reduce the risk of assessment and analytical bias, stringent blinding procedures are implemented for all outcome-related personnel without participant contact. Laboratory staff performing molecular analyses—including epigenetic, autophagy, and metagenomic assays—remain fully blinded to intervention assignments throughout sample processing and data acquisition. In addition, data management and statistical analyses are conducted by an independent team operating under complete blinding. Following final data download and database lock, intervention arms are anonymized using randomly generated labels (Group A, Group B, and Group C). The correspondence between these labels and the actual intervention groups is stored in an encrypted file under the exclusive custody of the Principal Investigator and is disclosed only after completion of all predefined primary and secondary statistical analyses.

Participants will begin the study no later than one week after the screening visit. A wave-based recruitment strategy will not be implemented, however no more than 25 patients will be included in any given month to facilitate follow-up.

### 2.13 Data collection, management and analysis

#### 2.13.1 Data collection

Data will be collected according to the trial scheme depicted in **Figure 1** using standardized instruments and electronic case report forms (CRFs). All assessors will undergo structured training to ensure consistency in administration and scoring. Duplicate measurements will be performed for key variables (e.g., anthropometry, blood pressure) to minimize measurement error. Randomization of sample placement in plates and duplicate readings will be applied for laboratory assays.

The principal investigator and study team will implement a comprehensive retention strategy to ensure participants complete all scheduled visits and maintain engagement throughout the study. Key actions include: i) establishing strong rapport with participants and fostering trust through consistent, personalized communication; ii) providing opportunities for participants and their families to ask questions and express concerns throughout the study, ensuring transparency and responsiveness; iii) promoting flexibility in scheduling the clinical visits; iv) reviewing study objectives and procedures during visits and offering brief Q&A sessions at each visit to confirm comprehension and address doubts; v) regularly assessing the likelihood of withdrawal and implementing tailored interventions—such as flexible scheduling, motivational support, and problem-solving strategies—to maintain interest and commitment. This approach prioritizes participant satisfaction, minimizes burden, and promotes adherence, thereby reducing attrition and ensuring high-quality follow-up data.

For any participant who discontinues the intervention or deviates from protocol, we will seek to collect a minimal outcome set within the allowable visit window (or remotely), prioritizing safety and core endpoints: weight and body composition, hepatic elastography, blood analyses and biological sampling. The discontinuation date, reasons for discontinuation, concomitant treatments, and any serious adverse event reports will be recorded in the ENSATI discontinuation form. Data from discontinuers will be included under intention−to−treat. If no in−person visit is possible, validated remote administration (telephone/video) will be used for questionnaires. All procedures follow predefined visit windows, with documentation of protocol deviations and imputation rules specified in the Statistical Analysis Plan.

#### 2.13.2 Data management

All study data will be recorded in secure electronic case report forms (eCRFs), complemented by paper forms when required. Data entry will be performed exclusively by trained personnel following standardized operating procedures to ensure accuracy and consistency. Each participant will be identified using a unique ENSATI study code assigned at the screening visit. This code will be used across all forms and databases, ensuring that no personal identifiers (such as name, address, or contact details) are linked to the study records. The linkage between participant identity and study code will be maintained in a separate, encrypted file accessible only to the principal investigator and authorized data managers. This approach guarantees participant confidentiality and compliance with data protection regulations. All data management procedures will comply with Good Clinical Practice (ICH-GCP) guidelines and applicable data protection regulations, including the EU General Data Protection Regulation (GDPR). Encryption, role-based access, and audit trails will be implemented to safeguard confidentiality and integrity throughout the study.

To ensure data quality and integrity, monthly quality control checks will be performed to detect potential anomalies, including missing data, values outside expected ranges, erroneous entries, illogical dates, inconsistencies across forms and study visits, and incomplete fields without valid justification. Any issues identified will be promptly reviewed and corrected to maintain accuracy and reliability. Details of the full Data Management Plan (DMP), including SOPs for data entry, validation, and query resolution, are available upon request and are referenced in the trial’s electronic repository.

#### 2.13.3 Data analysis

The full analysis pipeline is detailed in the Statistical Analysis Plan (SAP)(**Supplementary Material**). A complete variable dictionary, including variable descriptions, coded formats, data types, units, and classification schemes, has been developed to ensure transparency, reproducibility and to facilitate external validation efforts.

Continuous variables will be summarized as mean and standard deviation for normally distributed data or median and interquartile ranges for non-normal distributions, while categorical variables will be presented as counts and percentages. Baseline characteristics will be reported by intervention arm and compared using two-way ANOVA for continuous variables or χ² tests for categorical variables, applying non-parametric alternatives (Mann–Whitney U, Kruskal–Wallis, Fisher’s exact test) when assumptions are not met.

Statistical Analysis Hierarchy will follow a pre-specified hierarchy defined in the SAP (**Supplementary Material**). The primary analysis will follow the intention-to-treat (ITT) principle, including all randomized participants. The primary outcomes—changes from baseline to 6 months in body weight, segmental body composition (DXA), hepatic fat (CAP), and resting metabolic rate—will be evaluated using linear mixed-effects models (LMM). Models will incorporate intervention arm × time interaction as fixed effects and a subject-level random intercept to account for repeated measures and intra-cluster correlation. For these outcomes, no multiplicity adjustment will be applied, with significance set at p < 0.05. Secondary outcomes, including molecular biomarkers of aging (BoA), cardiometabolic markers, and microbiota, will be analyzed using similar LMM frameworks. To control the false discovery rate (FDR) arising from multiple testing, the Benjamini-Hochberg procedure will be applied (55). Exploratory analyses include multi-omics integration using machine learning algorithms (e.g., Random Forest, regularized regression) to identify mechanistic links between adherence and biological rejuvenation. Exploratory non-inferiority analyses will be conducted by comparing confidence intervals for treatment differences against predefined margins specified in the SAP (56).

A random forest–based imputation approach will be applied to handle missing data, as it is well suited to complex datasets that include both continuous and categorical variables and non-linear relationships. This method helps preserve the multivariate structure of the data and reduces bias, strengthening the reliability of downstream analyses (57).

The handling of missing data in the ENSATI trial formally operates under the Missing At Random (MAR) assumption, whereby the likelihood of missingness depends only on observed information rather than the missing values themselves. To maintain the integrity of the causal inference and avoid the artificial inflation of treatment effects, imputation will be strictly limited to confounding variables required for the hierarchical adjustment models (Models 1–5). Outcome variables (dependent), both primary and secondary, will remain unimputed in the main analysis to prevent the generation of artificial longitudinal trajectories. The imputation model will estimate missing values for relevant sociodemographic (e.g., education, employment), lifestyle (e.g., smoking, physical activity), and baseline clinical and dietary factors (e.g., BMI, comorbidities, diet adherence). The choice of a random forest method (such as missForest) is motivated by its non-parametric nature and its suitability for high-dimensional, mixed-type data typical in geroscience. It can capture complex, non-linear interactions without requiring predefined functional forms, and it leverages relationships across variables to improve accuracy. Uncertainty is addressed through the ensemble structure of the model, where predictions are averaged across many trees, helping reduce error and bias. To evaluate the robustness of the findings, sensitivity analyses will complement the imputed intention-to-treat analysis, including completers-only and per-protocol approaches. Additionally, E-values will be calculated to quantify how strong an unmeasured or poorly imputed confounder would need to be to fully explain away the observed treatment effects, providing further support for the study’s internal validity.

To isolate the intervention effect, a hierarchical adjustment strategy of five progressive models will be applied, ranging from unadjusted (Model 1) to a maximum adjustment model (Model 5) incorporating baseline weight, adherence, energy intake, and longitudinal changes in physical activity. When appropriate, random slopes will be added to account for individual variability in temporal trajectories.

To prevent overfitting, regularization techniques such as regularized regression will be applied, complemented by k-fold cross-validation and model selection based on AIC, BIC, and conditional AIC criteria (58, 59).Longitudinal correlation structures such as AR(1) or unstructured covariance will be used to appropriately model repeated measures. Diagnostic procedures will include residual and Q-Q plots, influence analyses, and graphical representations of trajectories and subgroup effects. Results will be reported as effect estimates with 95% confidence intervals, p-values, and adjusted q-values, complemented by visualizations such as forest plots and interaction graphs. Bayesian approaches may be explored for probabilistic interpretation of non-inferiority findings.

No formal auditing procedures or interim analyses are planned.

## 3 Results

### 3.1 Expected directions of the effect

**Figure 2:**
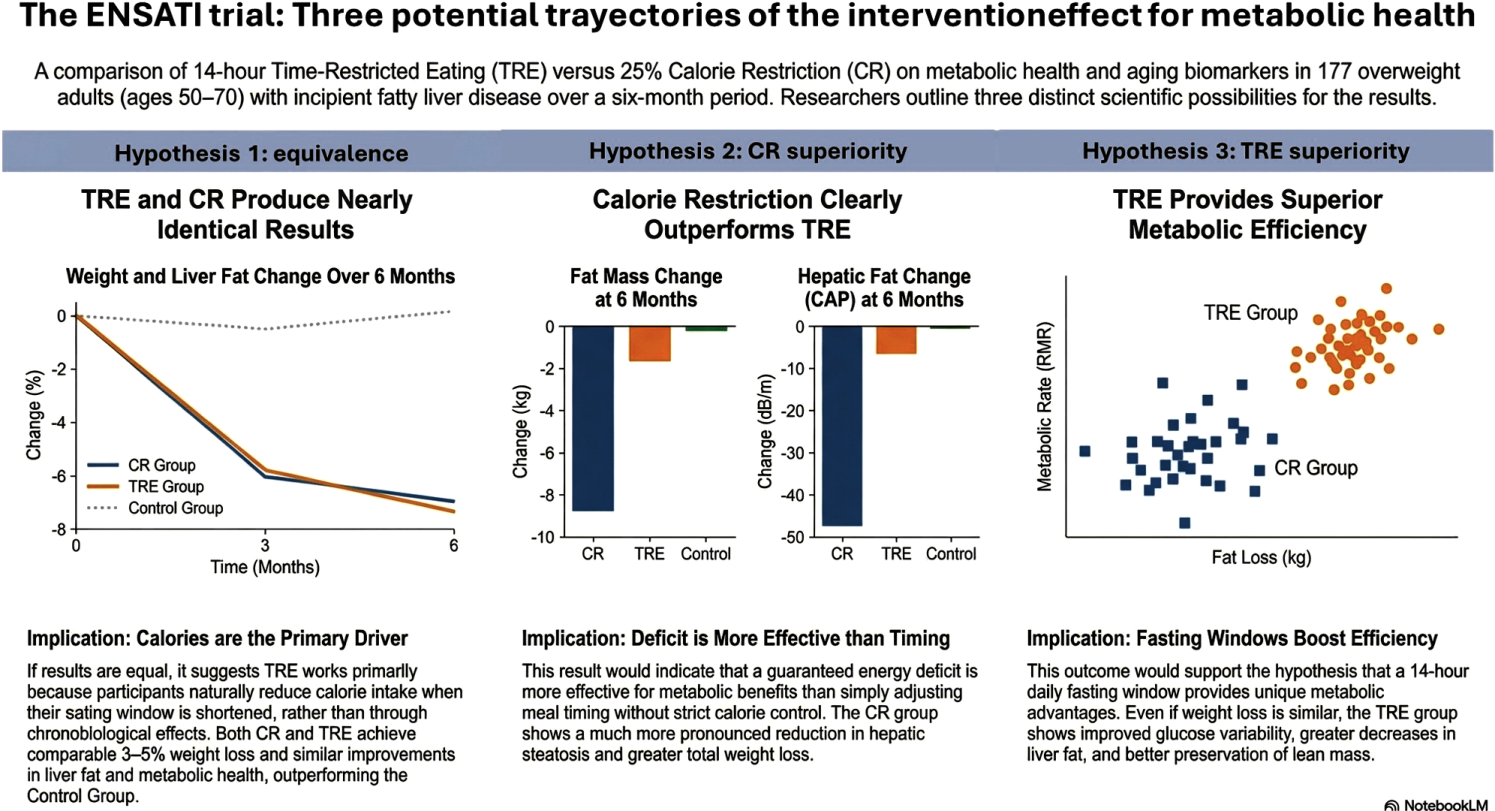
Hypothesized directions of effect for the ENSATI trial. This figure illustrates potential trajectories for primary outcomes, including body weight, hepatic fat (CAP), and resting metabolic rate across three intervention arms: Active dietary counseling control (CG), 25% Calorie Restriction (CRG), and 14:10 Time-Restricted Eating (TRE). Scenario 1 suggests equivalence, where both groups show similar weight and liver fat reduction, indicating TRE works via natural calorie restriction. Scenario 2 depicts CR superiority, with significantly larger reductions in fat mass and hepatic steatosis due to a guaranteed energy deficit. Scenario 3 proposes TRE superiority, highlighting enhanced metabolic efficiency, better liver fat reduction, and lean mass preservation despite similar weight loss. These scenarios are intended as a conceptual framework for comparing the relative contributions of energy restriction and chronobiological alignment.

The ENSATI trial is designed to evaluate three competing hypotheses regarding the relative effectiveness of 10-hour TRE and CR in improving metabolic health and molecular markers of aging compared to the active dietary counseling control group. Based on the study’s conceptual framework, three potential directions of effect are hypothesized for the primary outcomes (weight, hepatic fat, and metabolism):(**Figure 2**).

- Hypothesis 1 (equivalence/non-inferiority): it is hypothesized that CR and TRE may produce comparable clinical and molecular results. Under this framework, both groups would achieve similar improvements in body composition, liver fat, and metabolic health. Testing this hypothesis allows the trial to evaluate whether the benefits of TRE are primarily driven by the spontaneous reduction in energy intake that often accompanies a restricted eating window.
- Hypothesis 2 (CR superiority): alternatively, it is hypothesized that a prescribed 25% caloric restriction may result in significantly greater primary outcome improvements than TRE. This pathway tests whether a guaranteed, sustained energy deficit is the fundamental driver of metabolic health and molecular rejuvenation, suggesting that chronobiological alignment without controlled energy reduction offers limited additional therapeutic value.
- Hypothesis 3 (TRE superiority): Finally, the trial will evaluate whether 10-hour TRE (14:10) confers metabolic and molecular advantages that surpass those of a standard caloric deficit. This hypothesis posits that culturally adapted temporal alignment and cyclical nutrient deprivation trigger unique cellular responses—such as enhanced autophagic flux or epigenetic stabilization—that are independent of, or complementary to, energy restriction. Validation of this pathway would support the central geroscience hypothesis regarding the systemic benefits of nutritional chronobiology.

### 3.2 Strengths, limitations and contingency plans

The ENSATI protocol offers several strengths that set it apart from typical short-term nutritional interventions. Most studies in this field last only a few weeks, but ENSATI extends over an entire year, with six months of intervention followed by six months of follow−up. This longer duration makes it possible to evaluate not only immediate metabolic responses but also whether participants can sustain the changes and whether any molecular improvements truly persist. The study also incorporates high−resolution phenotyping techniques, including segmental DXA, indirect calorimetry, and hepatic elastography, which allow for a detailed understanding of how body composition, metabolic efficiency, and liver health evolve over time. Another distinctive feature is the integration of cutting−edge molecular biomarkers of aging, such as epigenetic clocks and measures of autophagic flux, linking traditional clinical outcomes with deeper biological mechanisms.

Despite these advantages, the protocol faces several challenges. First, the study is primarily powered for weight loss, instead of BoA. However, the alignment of our sample size with recent evidence—such as the study by Rigamonti et al. (n=72) on epigenetic age acceleration (59) and Espinoza et al. (n=30) on autophagy flux (60) —confirms that ENSATI is uniquely positioned to provide a high-resolution platform for the investigative exploration of secondary molecular outcomes. While the trial is formally powered for percentage weight loss, this sample size provides the resolution necessary to rigorously investigate the potentially subtle, yet mechanistically relevant, changes observed in secondary molecular BoA. By enrolling a cohort of this magnitude, ENSATI aims to move beyond standard pilot-scale geroscience trials toward a more substantive exploration of whether nutritional interventions can modulate the rate of biological deterioration. Second, maintaining adherence to a 0−hour eating window or a sustained 25% caloric deficit over many months can be difficult for participants. Third, in the TRE arm, reduced calorie intake may occur unintentionally. Although we applied strict protocols to assess dietary intake and to track changes in calorie intake across groups, this was not a fully controlled feeding trial in which all meals were provided. Consequently, some degree of measurement error and residual confounding related to self−reported intake cannot be completely ruled out, particularly in a free−living setting. While this design choice enhances external validity, it limits our ability to fully disentangle the independent effects of meal timing from unintentional calorie restriction. Additionally, substantial variability between individuals may introduce noise into molecular datasets. Finally, the 12-month duration of the ENSATI trial, while a strength for assessing durability, introduces significant risks regarding participant burden and long-term adherence. We acknowledge that maintaining a strict 10-hour eating window or a 25% caloric deficit for six months is a demanding task that may lead to substantial non-compliance or dropout. Fourth, a significant limitation of this protocol is the reliance on peripheral blood mononuclear cells (PBMCs) as a systemic surrogate for biological aging, which introduces several technical and biological challenges. Autophagic flux measurements in PBMCs are highly sensitive to pre-analytical delays; storage of whole blood at room temperature or on ice for as little as four hours has been shown to unpredictably alter LC3B-II levels, necessitating immediate processing to ensure data validity. Second, the high inter-individual variability observed in human PBMC autophagy can be exacerbated by a participant’s most recent food intake, as these cells are acutely responsive to nutrient signaling (e.g., insulin and amino acids). To mitigate this, the ENSATI trial enforces a strict 10-hour overnight fast prior to collection. Furthermore, because the PBMC fraction is a heterogeneous mix of lymphocytes and monocytes, variations in cell-type composition between participants may introduce noise into the autophagic flux signal. Finally, caution is required when generalizing these findings, as autophagic responses in blood cells may not consistently reflect the aging status or metabolic adaptations of other tissues, such as cardiomyocytes or hepatic cells (61, 62).

To address anticipated issues, the protocol includes multiple troubleshooting strategies. Missing data are mitigated using random−forest imputation and an intention−to−treat approach. Because dietary self−reports can be unreliable, objective tools such as three−day food records, time−stamped meal logs, and continuous glucose monitoring help verify adherence and metabolic responses. Additionally, our statistical analysis plan includes a fully adjusted Model 5 that incorporates longitudinal changes in energy intake. This approach, together with the use of three−day dietary records and time−stamped food diaries, is designed to account for potential confounding and to explore the relative contributions of meal timing, assessing whether the observed benefits of TRE remain evident after statistical adjustment for the observed caloric reduction. Frequent follow−up contacts aim to detect barriers early and prevent protocol deviations. To safeguard statistical power, the study is powered by 177 participants, incorporating an anticipated 20% dropout rate based on previous longitudinal geroscience trials. However, if attrition exceeds this threshold, the study’s internal validity could be compromised. To mitigate this, we have implemented a ‘completers-only’ sensitivity analysis to evaluate the robustness of our findings in those who successfully finished the protocol. Finally, laboratory and clinical measurements are standardized through randomized plate assignment, duplicate assays, and a consistently trained assessment team, reducing technical variability and ensuring data quality.

## 4 Discussion

Healthy aging is a multidimensional process influenced by diet quality, lifestyle, and metabolic factors. A recent 30-year prospective study in over 105,000 U.S. adults demonstrated that adherence to healthy dietary patterns—particularly the Alternative Healthy Eating Index—substantially increased the odds of healthy aging, defined as survival to ≥70 years without major chronic disease and with preserved cognitive, physical, and mental health (16). Beyond diet composition, emerging evidence highlights the role of calorie intake and meal timing in modulating aging processes. In our previous work within the PREDIMED-Plus trial, a 3-year lifestyle intervention combining a 25% energy-reduced Mediterranean diet with structured physical activity led to significant improvements in vascular and immune aging markers (13).

Another promising approach to increase health span is fasting. The study of fasting’s impact on biological aging, particularly body composition and cardiometabolic risk factors, is an emerging and promising field. Recent trials have examined time-restricted eating (TRE) in various formats. A pilot RCT in adults with obesity found comparable weight loss with 12- and 14-hour fasting, but fasting glucose improved only with the longer window (63). Early TRE aligned with circadian rhythms (06:00–15:00) for 5 weeks enhanced insulin sensitivity, glycemic control, inflammation, and gut microbiota diversity compared with mid-day TRE (24). In contrast, a 12-month trial reported no added benefit of TRE combined with calorie restriction versus calorie restriction alone (64). Short-term studies suggest modest improvements in weight, blood pressure, and lipids, though findings are inconsistent and lean mass loss has been observed in some cases (22, 23). A large multicenter RCT showed similar weight loss across early, late, and self-selected TRE, with only slight glucose advantages for early TRE (25). Supporting this, a scoping review of 19 RCTs found intermittent fasting reduced weight and visceral fat without excess muscle loss in adults ≥45 years, though data in those ≥70 years are scarce (65).

Despite these promising findings, important limitations must be acknowledged. The existing scientific evidence suffers from several methodological constraints that limit definitive conclusions regarding TRE and CR for healthy aging (1): intervention periods are typically short (most are ≤ 12 weeks), capturing only acute adaptation rather than long-term efficacy or maintenance; (2) studies are generally small in scale,; and (3) there is a consistent failure to integrate comprehensive molecular BoA alongside traditional anthropometric measures. The ENSATI trial establishes an integrative investigative framework designed to address these critical gaps. Rather than introducing novel analytical techniques, the trial integrates established clinical phenotyping with advanced molecular biomarkers into a 12-month longitudinal design. This structure is intended to provide a high-resolution platform for exploring the relative contributions of energy restriction and chronobiological alignment. The trial protocol utilizes a randomized, controlled parallel-group design, incorporating blind molecular and statistical assessments, over a significantly extended period, incorporating a high-resolution analysis of both physiological and advanced molecular outcomes. The inclusion of three rigorously controlled parallel arms—an active dietary counseling control (CG), gold-standard 25% caloric restriction (CRG), and a culturally adapted 14:10 TRE group— facilitates a more rigorous exploration of the potentially distinct roles of energy restriction-dependent versus chronobiological-dependent effects on hallmarks of aging within a high-risk population.

Previous high-impact pilot trials in TRE have been limited by sample size: for instance, the study by Peeke et al. involving 14:10 TRE enrolled only 78 participants, with 60 completing the 8-week intervention (63). Similarly, a comparative study of early and mid-day TRE regimens by Xie et al. included only 90 randomized participants, with 82 completing the 5-week trial in a healthy, non-obese population (24). The ENSATI trial addresses this challenge directly by aiming to enroll 177 participants. This robust sample size is intended to provide the resolution necessary to rigorously explore the potentially smaller, yet mechanistically relevant, changes observed in molecular BoA. By establishing this comparative and investigative framework, the trial aims to move beyond pilot-scale observations toward a more substantive exploration of whether nutritional interventions can modulate the rate of biological deterioration in high-risk populations.

Another major constraint across the field of nutritional trials focusing on fasting is the short duration of most published RCTs. Foundational trials such as the TREAT trial (66), the study by Dote-Montero et al. (25), and the earlier pilot studies (24, 63) typically lasted 12 weeks or fewer. Such short-term interventions capture only acute responses and early adaptations, offering limited insight into long-term sustainability or durability of benefits. The ENSATI trial addresses this gap with a 12-month design comprising a 6-month active intervention and a 6-month post-intervention follow-up without counseling. This structure allows evaluation of long-term adherence and translational potential of calorie restriction and TRE, specifically assessing whether molecular and physiological benefits—particularly biological age and hepatic fat—are maintained once structured support ends, informing evidence-based, sustainable health span guidelines.

Another primary unresolved question in TRE research is whether its benefits are derived primarily from the chronobiological alignment (timing of food intake) or simply from an unintended reduction in total energy intake. Previous randomized designs have often confounded these variables: the TRE arm in the Lowe et al. study, for example, involved *ad libitum* eating without caloric guidance (66). Conversely, the Liu et al. study ensured caloric restriction in both the TRE and continuous eating groups, ultimately attributing weight loss almost entirely to the caloric deficit (64). The ENSATI trial employs a superior mechanistic design with three parallel arms: active dietary counseling control CG that receives general Mediterranean-style dietary advice without mandated energy restriction; 25% CRG designed for maximal metabolic impact via energy deficit; and the TRE group that implements a 14:10 window with encouragement to follow a Mediterranean-style diet without explicit energy restriction. The design allows for a direct, high-powered comparison of the effects of TRE versus a calorically equivalent intervention (CRG) and a minimally treated control (CG).

Rigorous control and monitoring of caloric intake across all intervention arms is paramount to resolve the major confounding issue of energy deficit in TRE research. The ENSATI protocol mandates detailed dietary assessment in all three groups. Critically, adherence in both the TRE and CG arms is monitored through detailed dietary diaries, requiring participants to record the timing of all meals and completing three-day dietary records (two weekdays, one weekend day) at baseline, mid-term (3 months), and end-of-intervention (6 months). This methodology is designed to mitigate the acknowledged challenge that ad libitum TRE frequently results in an unintentional caloric reduction simply due to the shortened eating window. The detailed monitoring of actual energy intake enables the trial to explore the relative contributions of timing versus energy deficit on the observed outcomes. This comparative assessment is essential for investigating whether TRE offers physiologically distinct advantages beyond its role as a potential strategy for spontaneous calorie reduction.

The most distinctive methodological strength of the ENSATI trial is the rigorous, large-scale integration of molecular BoA. This comprehensive assessment shifts the focus from treating symptoms of chronic disease to intervening directly in the underlying processes of biological aging, thus aligning the study precisely with the forefront of geroscience. The ultimate metric of a successful anti-aging intervention is its ability to decelerate the rate of biological aging. However, most existing TRE and CR trials rely predominantly on traditional anthropometric and clinical chemistry measures, which are surrogates for overall health and fail to capture the complex molecular cascade that underpins longevity. By integrating BoA, ENSATI is equipped to assess the fundamental hypothesis of geroscience: that nutritional interventions can modulate the rate of biological deterioration.

Despite its high-resolution design, the ENSATI trial is subject to several methodological challenges that warrant a cautious interpretation. The reliance on self-reported dietary data remains a significant limitation, as recall bias and under-reporting can introduce noise when attempting to quantify exact energy intake. Moreover, because TRE often leads to unintentional caloric reduction, an alternative interpretation of our findings could be that any observed molecular rejuvenation is driven primarily by spontaneous weight loss rather than chronobiological alignment. To critically address these factors, our analytical framework incorporates mediation models and a maximum adjustment model (Model 5) to statistically account for longitudinal shifts in energy intake. By acknowledging these complexities, the trial is positioned not as a claim of definitive isolation of effects, but as a rigorous exploration of the relative contributions of timing and deficit within a real-world, free-living population.

In summary, the ENSATI study presents a prospective trial framework designed to investigate key methodological gaps in nutritional geroscience. By implementing this multi-arm design, the protocol provides a structure for the systematic exploration of how meal timing and energy intake influence health span, aiming to generate evidence-based insights for future geriatric nutritional guidelines. By implementing a multi-arm design over a 12-month period with a target of 177 participants, the protocol facilitates a rigorous exploration of the relative contributions of meal timing and energy intake on health span. The trial integrates advanced physiological phenotyping—including segmental DXA, indirect calorimetry, and hepatic elastography—with high-resolution molecular biomarkers of aging (BoA). This integrative approach establishes a structure to investigate the central chronobiological hypothesis: whether culturally adapted temporal alignment and cyclical nutrient deprivation trigger molecular and metabolic responses that differ from or complement those induced by a standardized caloric deficit. By comparing these interventions against an active dietary counseling control, ENSATI aims to provide evidence-based insights into the potential of nutritional chronobiology to decelerate biological deterioration in high-risk populations.

## Data Availability

The original contributions presented in the study are included in the article/**Supplementary Material**. Further inquiries can be directed to the corresponding author.
