# Supplementary material for: Time-restricted eating versus calorie restriction for improving biomarkers of age in adults with overweight or obesity and incipient fatty liver disease: protocol for the ENSATI randomized controlled parallel groups trial
Source: Front Endocrinol (Lausanne). 2026 Jun 12;17:1849550. doi: 10.3389/fendo.2026.1849550 (PMC13303218; doi:10.3389/fendo.2026.1849550)
Supplement: Supplementary file 1 [file DataSheet1.docx]

ENSATI STUDY: STATISTICAL ANALYSIS PLAN

# **Administrative information**

**Title and registration**

Healthy aging through time- restricted eating in adults with overweight/obesity and incipient liver disease: the ENSATI study.

This study has been prospectively registered in ClinicalTrials.gov (<https://clinicaltrials.gov/study/NCT05880095?term=ENSATI&viewType=Card&rank=1>) with number NCT05880095 and registration date 18th of May of 2023.

**Version**

SAP – version 2- date 08/05/2025

Protocol - version 4 – date 19/04/2024

Available in: <https://ensati.wixsite.com/ensati>

**SAP amendments**

- Extended description of imputing methodology

- Change the trial design from “single-blind” to “open-label”

**Roles and responsibilities**

**1.Person who writes the SAP**

José Antonio Celada Guerrero

Nutritional Control of the Epigenome Group. Precision Nutrition and Obesity Program. IMDEA Nutrition.

**2. Responsible of Statistical Analyses**

Víctor de la O Pascual, PhD

Nutritional Control of the Epigenome Group. Precision Nutrition and Obesity Program. IMDEA Nutrition.

**3. Principal Investigator**

Lidia Daimiel Ruiz, PhD

Nutritional Control of the Epigenome Group. Precision Nutrition and Obesity Program. IMDEA Nutrition.

CIBER Physiopathology of Obesity and Nutrition

Content

[**1.** **Administrative information** 1](#_Toc227765460)

[**2.** **Abstract** 5](#_Toc227765461)

[**3.** **Introduction** 6](#_Toc227765462)

[a. State-of-the-art and rationale 6](#_Toc227765463)

[b. Objectives 7](#_Toc227765464)

[**4.** **Methods** 8](#_Toc227765465)

[a. Study design 8](#_Toc227765466)

[b. Population description 9](#_Toc227765467)

[c. Sample size 10](#_Toc227765468)

[d. Randomization 11](#_Toc227765469)

[e. Intervention arms 11](#_Toc227765470)

[f. Blinding 12](#_Toc227765471)

[g. Study variables 12](#_Toc227765472)

[i. Primary variables 12](#_Toc227765473)

[ii. Secondary variables 12](#_Toc227765474)

[iii. Confusing variables 13](#_Toc227765475)

[**5.** **Conceptual framework and analytical pipeline** 14](#_Toc227765476)

[a. Analytical scenarios 14](#_Toc227765477)

[b. Analytical approach 15](#_Toc227765478)

[i. Intention to treat analyses 15](#_Toc227765479)

[ii. Per protocol analyses 15](#_Toc227765480)

[iii. As per treatment analyses 16](#_Toc227765481)

[iv. Stratified analyses 16](#_Toc227765482)

[v. Adjustment strategies 17](#_Toc227765483)

[c. Temporal framework of the analyses 19](#_Toc227765484)

[**6.** **Statistical principles** 21](#_Toc227765485)

[a. Treatment of variables 21](#_Toc227765486)

[b. Descriptive analyses of the population 22](#_Toc227765487)

[c. Missing data treatment 22](#_Toc227765488)

[d. Confidence intervals and p value 22](#_Toc227765489)

[i. Primary outcomes 22](#_Toc227765490)

[ii. Secondary outputs 23](#_Toc227765491)

[7. **Methods to evaluate the effect of the intervention** 23](#_Toc227765492)

[a. Main model 23](#_Toc227765493)

[b. Sensitivity analyses 24](#_Toc227765494)

[c. Correlation matrix 24](#_Toc227765495)

[d. Mediation analyses 25](#_Toc227765496)

[**8.** **Omics data analysis: metagenomics and epigenomics** 25](#_Toc227765497)

[a. Epigenomic analyses (DNA Methylation) 26](#_Toc227765498)

[b. Faecal Metagenomics Analyses 26](#_Toc227765499)

[c. Integration with longitudinal analyses 26](#_Toc227765500)

[**9.** **Graphical representation** 27](#_Toc227765501)

[a. Tables 27](#_Toc227765502)

[b. Figures and graphical representations 28](#_Toc227765503)

[10. References 28](#_Toc227765504)

# **Abstract**

The ENSATI trial addresses the challenge of population ageing by focusing on maximizing the healthy lifespan through strategic nutritional interventions. This study is a randomized, controlled, parallel‑group clinical trial involving 177 adults aged 50 to 70 years with overweight and early-stage fatty liver disease. Participants are allocated to three arms: time‑restricted eating (TRE 14:10), traditional 25% caloric restriction (CR), and an active dietary counselling control group following a standard Mediterranean diet. The design spans a total of 12 months, comprising a 6‑month active intervention phase followed by a further 6‑month post‑intervention follow‑up to assess the durability of effects.

The primary objective is to evaluate changes in body weight, body composition (fat and lean mass), liver fat, and basal metabolism. As secondary objectives, the study integrates high‑resolution biomarkers of ageing (BoA), such as epigenetic clocks, autophagic flux, and immunosenescence, and analyses faecal microbiota and cardiometabolic health using continuous glucose monitoring.

Methodologically, analyses follow the intention‑to‑treat principle using linear mixed‑effects models with repeated measures. A hierarchical adjustment strategy with five models is applied to account for sociodemographic, clinical, and lifestyle factors and to isolate the true dietary effect. To ensure robustness, random‑forest‑based imputation, “completers‑only” sensitivity analyses, and E‑value calculations are employed to assess the impact of potential unmeasured confounders. Artificial intelligence and machine‑learning approaches are also used to explore mechanistic links between molecular patterns and adherence. This trial aims to determine whether the chronobiological alignment of TRE confers metabolic benefits independent of energy deficit, transforming complex omics data into clinical guidance to promote healthy ageing.

# **Introduction**

## State-of-the-art and rationale

**Global Context and Geroscience**

The unprecedented increase in global life expectancy has shifted the primary goal of geroscience: the focus is no longer merely on extending lifespan, but on maximizing the period of healthy life, or “health span” (1). Biological ageing is defined as a dynamic and individualized process of physiological decline, influenced by a complex interplay of intrinsic (genetic, metabolic) and extrinsic factors, among which diet stands out as the most relevant modifiable behavioural factor (2). In this context, nutritional interventions represent a practical and scalable strategy to mitigate age‑related vulnerability, morbidity, and mortality (1).

**Limitations of the Current Evidence**

Although dietary patterns such as the Mediterranean Diet have demonstrated benefits for vascular ageing markers and immunosenescence, the specific impact of caloric restriction (CR) (3,4) and intermittent fasting in humans remains under debate (5,6). Strategies such as time‑restricted eating (TRE) have gained popularity by shifting the focus from how much to eat to when to eat, aiming to align food intake with circadian rhythms (6–8). However, the current scientific literature presents three critical limitations that the ENSATI study seeks to address:

- Insufficient duration: Most previous randomized controlled trials last ≤12 weeks, capturing only acute adaptations rather than the sustainability or durability of molecular changes.
- Limited scale and power: Many studies involve small sample sizes, resulting in high heterogeneity and insufficient statistical power to detect subtle changes in molecular biomarkers.
- Mechanistic confounding: There is a lack of clarity as to whether the benefits of TRE arise from chronobiological alignment or from an unintentional reduction in energy intake.

**Rationale for the ENSATI Trial**

The ENSATI trial has been designed as a 12‑month, randomized, controlled, open-label clinical trial to overcome these gaps, evaluating 177 adults with overweight and early‑stage fatty liver disease. The study directly compares three arms: an active dietary counselling control group (usual care), a 25% caloric restriction group (CRG), and a TRE 14:10 group. This design allows for the disentangling of energy‑dependent versus chronobiology‑dependent effects on the hallmarks of ageing.

**Need for a Robust Statistical Analysis Plan (SAP)**

The high interindividual variability inherent in nutritional trials necessitates a rigorous analysis plan to identify biologically meaningful differences. The ENSATI SAP ensures transparency and reproducibility with the use of linear mixed‑effects models to assess longitudinal changes, adjusting for key confounders such as physical activity, baseline adherence, and actual energy intake. In addition, the integration of high‑resolution biomarkers of ageing (BoA)—including epigenetic clocks, autophagic flux, and metagenomics—requires advanced imputation techniques and correction for multiple testing to transform complex molecular data into definitive clinical evidence. Ultimately, this analysis will provide evidence‑based guidance to optimize metabolism and functional health in an ageing, metabolically compromised population.

## Objectives

The objectives of the Statistical Analysis Plan (SAP) for the ENSATI trial are designed to provide a technical and pre‑specified roadmap that ensures the transparency, reproducibility, and scientific validity of the study findings.

1. **Primary Objective: Evaluation of Clinical Efficacy:**

The primary aim is to formally compare the effects of time‑restricted eating (TRE 14:10) versus traditional caloric restriction (CR 25%) and an actively dietary counselling control group on the primary outcomes after 6 months of intervention. Specifically, the analysis seeks to quantify changes in:

- Body weight and segmental body composition (fat mass and lean mass assessed by DXA).
- Hepatic fat content (measured by elastography/FibroScan).
- Basal metabolic rate (assessed by indirect calorimetry).

1. **Secondary Objectives: Impact on Biological Ageing and Metabolic Health**

The SAP defines the methods to analyse a broad range of markers that characterize the “health span”:

- **Biomarkers of Ageing (BoA):** Assessment of effects on epigenetic clocks, autophagic flux, and markers of immunosenescence.
- **Cardiometabolic Health and Microbiome:** Analysis of 24‑hour glucose curves (CGM), lipid profile, and changes in faecal microbiome diversity and function using metagenomics.
- **Psychosocial and Cognitive Well‑being:** Evaluation of changes in sleep quality, cognitive functions (attention, memory), anxiety, and overall well‑being.

1. **Durability and Long‑Term Follow‑up Objective:**

A key objective is to determine whether improvements observed after the 6‑month active intervention phase are maintained or return to baseline levels after an additional 6 months of follow‑up without direct counselling (V₁₂). This will allow the assessment of the real‑world sustainability of the nutritional interventions.

1. **Methodological and Safety Objectives**

- **Intention‑to‑Treat (ITT) and Sensitivity Analyses:** Ensure that all randomized participants are included in the primary analysis to avoid selection bias, complemented by per‑protocol analyses to assess efficacy among highly adherent participants.
- **Subgroup and Stratified Analyses:** Investigate whether the interventions exert differential effects according to sex or age range.
- **Error Control and Data Quality:** Implement the Benjamini–Hochberg procedure to correct for multiple comparisons and apply robust missing‑data imputation techniques (Random Forest) to preserve statistical power.
- **Safety Assessment:** Analyse the incidence of reported adverse events (fatigue, mood changes, gastrointestinal disturbances) across the three intervention arms.

1. **Exploratory Non‑Inferiority Analysis:**

The SAP also considers the possibility of conducting analyses to determine whether TRE is non‑inferior to CR in terms of metabolic benefits, which would suggest that modifying meal timing may represent a valid alternative to traditional caloric reduction for improving health in older adults.

# **Methods**

## Study design

The ENSATI study is an exploratory, randomized, controlled, parallel-group clinical trial with a open-lable design conducted in Madrid, Spain. The protocol includes 177 adults aged 50 to 70 years with overweight or obesity and incipient fatty liver disease. Participants are randomly assigned to one of three intervention arms: an active dietary counselling control group (CG), a traditional 25% caloric restriction group (CRG), and a time-restricted eating (TRE) group with a 14-hour fasting window and a 10-hour eating window. The study design comprises a six-month intervention phase, followed by a further six-month post-intervention follow-up period to assess the durability of the effects without direct counselling. The protocol has been developed in accordance with the SPIRIT guidelines, and results will be reported following CONSORT guidelines, with primary outcomes focusing on changes in body weight, body composition, liver fat, and basal metabolism.

## Population description

The ENSATI study population consists of 177 adults, both men and women, aged between 50 and 70 years. Participants are recruited in the Community of Madrid, Spain, primarily through primary health care centres.

The specific eligibility criteria include:

- Having overweight or obesity, defined by a Body Mass Index (BMI) between 25 and 40 kg/m².
- Having early-stage fatty liver disease, validated by a Fatty Liver Index (FLI) equal to or greater than 30.
- Maintaining a daily eating window of at least 12 hours, stable sleep patterns, and a body weight that has not fluctuated significantly in the three months prior to study initiation.

The study design aims to maintain a 1:1 sex ratio and uses stratified randomization by sex and age range (50–59 and 60–70 years) to ensure comparability between groups. Individuals using weight-loss medications, participating in other lifestyle intervention programs, or presenting health conditions that could compromise their safety or adherence to the protocol are excluded.

The following table presents the detailed inclusion and exclusion criteria:

| **Inclusion Criteria** | **Exclusion Criteria*** |
| --- | --- |
| • Men and women • 50–70 years old • Body Mass Index (BMI) 25–40 kg/m² • Presence of fatty liver disease confirmed by ultrasound or biomarkers (Fatty Liver Index > 30 when ultrasound is unavailable) • Usual daily eating window at baseline ≥ 12 hours • Regular sleep cycle in the month prior to enrolment (7 ± 2 hours/day) • Stable weight during the preceding 3 months (≤ 4 kg variation) • No plans to modify physical activity level during the 6‑month study period • Not currently following a weight‑loss diet | • Failure to meet inclusion criteria • Women of reproductive age without established menopause (no menstrual period for the previous 12 months) • Excessive alcohol consumption (CAGE score > 2) • Smokers who changed smoking habits in the 6 months prior to enrolment • Diagnosed renal disease • Prevalent cardiovascular disease or angina pectoris • Liver disease other than non‑alcoholic fatty liver disease • Uncontrolled endocrine disease (hypo/hyperthyroidism, type 1 or type 2 diabetes, adrenal disorders)* • Uncontrolled hypertension • Pancreatitis • Medical treatment affecting weight, appetite, energy expenditure, or sleep in the past 3 months • Food allergies or intolerances that prevent adherence to the intervention protocol • Diagnosed eating behaviour disorders • Shift workers • Participation in another study that may interfere with the present protocol • Social, cultural, or psychological factors limiting adherence (e.g., inability to consume solid foods, unstable residence, institutionalization) |

## Sample size

The variable used for the sample size calculation is the percentage of weight loss relative to baseline. This variable was chosen based on previous studies consistently showing that participants undergoing a time-restricted eating (TRE) intervention reduce their body weight by approximately 3% (6,9,10).

Our interventional clinical trial will follow the design of an open-label, randomized, controlled, parallel-group trial, in which a two-way ANOVA will be used to compare changes in study variables across the three intervention arms. The sample size calculation considers three intervention groups, a 5% difference in weight loss between groups, and a within-group variability in weight loss of 50%.

We assume a significance level of 0.05 (type I error) and a statistical power of 80% (type II error). The sample size calculation was performed using the power.anova.test function in R version 4.1.3 (2022-03-10) with the RStudio software. Based on these parameters, 49 participants per group are required. Assuming a 20% loss to follow-up, 59 participants per group are needed to achieve statistical significance for the proposed parameter.

Therefore, a total of 177 participants will be recruited, with an approximate sex ratio of 1:1.

The choice of percentage weight loss as the primary powering variable is justified by its nature as a traceable trait directly linked to biological aging . Currently, there is a lack of reference trials in similar populations whose sample size calculations are based on molecular BoA. However, our choice is supported by preliminary findings from a similar cohort (n=116) where significant improvements in immunosenescence and HDL functionality were detected with a smaller sample size (16). Thus, our target of 177 participants provides more than adequate power for molecular secondary outcomes.

This sample size, while formally calculated for percentage weight loss, is intended to provide the resolution necessary to rigorously investigate the potentially subtle, yet mechanistically relevant, changes observed in secondary molecular BoA. By enrolling a cohort of this magnitude, ENSATI moves beyond standard pilot-scale geroscience trials to explore whether nutritional-induced weight loss is accompanied by significant shifts in molecular hallmarks of aging

## Randomization

Participants will be randomly assigned to the different intervention arms using a complete block randomization method. The randomization blocks will be sex (male/female) and age range (50–59 years / 60–70 years). This approach ensures that all groups are comparable in terms of sex and age. Participants belonging to the same household will be identified, and a cluster randomization approach will be applied to assign family units to the same intervention arm. Participants will be randomly allocated to one of the three intervention arms (CG, CRG, or TRE) using a parallel-group design with an equal allocation ratio (1:1:1). Variable block sizes (ranging from 4 to 16 participants) will be used according to the monthly recruitment rate to maintain allocation concealment and prevent predictability in group assignment. The randomization strategy has been designed to ensure allocation concealment and balance across groups. Randomization will be conducted using the randomizr package in R. No member of the research team responsible for delivering the intervention or monitoring participants will have access to the randomization blocks or the randomizr script.

## Intervention arms

The intervention will be open-label, as each participant will be informed of the group to which they have been assigned at visit V_00. The total duration of the intervention will be 6 months. The intervention for each group will be as follows:

• **TRE group**: Participants will follow a Mediterranean-style diet with no energy restriction during an allowed eating period of 10 hours, leaving a 14-hour fasting window. Participants may choose the 10-hour eating window that best fits their habits, although they will be given a selection range between 06:00 and 20:00. During the fasting period, water and other non-caloric beverages will be permitted. The TRE regimen must be followed on both weekdays and weekends.

• **CR group:** Participants will follow a Mediterranean-style diet with a 25% caloric restriction and no restriction on eating times. To implement caloric restriction, appropriate energy intake will be calculated according to current body weight using the Harris–Benedict formula, adjusted for physical activity level. From the calculated intake, 25% will be subtracted. A Mediterranean-style menu with portion sizes ensuring the defined intake for each participant will be developed. Existing materials from the PREDIMED-Plus trial will be used.

• **DMed group:** Participants will be allowed to follow their usual diet, with no restriction on eating times. However, due to their condition of overweight or obesity, they will be given general recommendations to follow a healthy Mediterranean-style dietary pattern.

## Blinding

The study follows a open-label design. Participants will be informed of their assigned intervention arm during the baseline clinical visit. All personnel involved in delivering the nutritional interventions, conducting clinical and phenotypic interviews, performing anthropometric measurements, assessing body composition, liver elastography, and indirect calorimetry will also be aware of each participant’s assigned arm. Likewise, staff responsible for fitting accelerometers and continuous glucose monitoring devices will be informed of group allocation.

In contrast, strict masking procedures will be maintained for all personnel responsible for data analysis. Participants will begin the study no later than one week after the screening visit. No wave-based recruitment strategy will be implemented; however, no more than 25 patients will be enrolled in a single month to facilitate follow-up.

## Study variables

For further detail, the variable dictionary file is included as an annex to this plan. This file contains the name of each variable, its type (numeric, categorical, or character), its labels, and its mode of handling (**Annex I**).

### Primary variables

They focus on the changes occurring from the start of the study to 6 months in the following areas:

• **Body weight and body composition**: Including weight (kg), height (cm), body mass index (BMI, kg/m²), fat mass (% and kg), lean mass (%), muscle mass (% and kg), and visceral fat index, assessed by dual-energy X-ray absorptiometry (DXA) and bioelectrical impedance.

• **Liver fat**: Measured by elastography (FibroScan) to determine the degree of steatosis and fibrosis (CAP and dB).

• **Metabolism**: Specifically, resting metabolic rate (RMR) determined by indirect calorimetry (kcal/day).

### Secondary variables

These variables assess the multidimensional impact of the nutritional interventions:

• **Cardiometabolic markers:** Fasting glucose (mg/dL), glycated haemoglobin A1c (HbA1c, %), complete lipid profile (total cholesterol, HDL, LDL, triglycerides; mg/dL), liver function (ALT, AST, and GGT; U/L), renal function (glomerular filtration rate, %), and systolic and diastolic blood pressure (mmHg).

• **Dynamic glycaemic control**: 24-hour glucose curves and glycaemic variability assessed by continuous glucose monitoring (CGM).

• **Hepatic indices**: Fatty Liver Index (FLI), Hepatic Steatosis Index (HSI), and Fibrosis Index (FIB-5).

• **Molecular biomarkers of aging (BoA):** Second- and third-generation epigenetic clocks (based on DNA methylation), autophagic flux, and markers of immunosenescence (CD28⁻ T lymphocytes as a percentage of the total lymphocyte population).

• **Microbiota**: Diversity, composition, and functionality of the faecal microbiota assessed by metagenomic sequencing.

• **Sleep and circadian rhythms**: Sleep quality (PSQI index), chronotype (MEQ questionnaire), and objective parameters assessed by accelerometry.

• **Psychological and cognitive health:** Anxiety levels (Hamilton Anxiety Rating Scale), emotional eating (emotional eating questionnaire), mood state (EVEA questionnaire), general well-being (W-BQ12 questionnaire), perceived health status (SF-36 questionnaire), and verbal memory and selective attention (Rey Auditory Verbal Learning Test and the STROOP colour test).

• **Eating behaviour**: Adherence to the Mediterranean diet (MEDAS), energy intake, and macronutrient composition.

### Confusing variables

For the final statistical models, the following potential confounding factors will be considered:

• **Sociodemographic factors:** Age (years), sex, educational level and years of schooling, marital status (married, divorced/separated/single), employment status (employed, temporarily inactive, or permanently inactive), place of origin (rural/urban), and number of people sharing the household.

**• Lifestyle factors**: Smoking status (current smoker, former smoker, or never smoker) and physical activity (measured in METs/min/week and classified into light, moderate, and vigorous activity levels, assessed at baseline and their longitudinal changes).

• **Clinical factors:** Medication use (type of medication and daily dose) and the prevalence (Yes/No) of hypertension, diabetes, and depression.

• **Dietary factors:** Baseline adherence to the Mediterranean diet (MEDAS questionnaire score) and nutritional profile (derived from dietary surveys, including macro- and micronutrients expressed as % and g/day).

Regarding dietary variables considered as potential confounders, adherence to the Mediterranean diet will only be considered at baseline, assessed using the MEDAS questionnaire score. This is because the Mediterranean diet constitutes part of the intervention itself, as all three study arms are based on this dietary pattern; therefore, subsequent differences in adherence are not considered a confounding factor, but rather an intrinsic component of the intervention.

In contrast, energy intake will be analysed both at baseline and in terms of its changes throughout the intervention, with the aim of isolating the specific effect of the different intervention strategies on the primary study outcomes. This approach is particularly relevant in the case of time-restricted eating, as it allows discrimination of whether the observed effects are attributable to the temporal pattern of intake independently of potential changes in total energy consumption.

# **Conceptual framework and analytical pipeline**

## Analytical scenarios

To complete the expected outcome scenarios of the ENSATI trial, based on its three-arm design and the central study hypothesis, the scenarios are defined as follows:

• **Non-inferiority scenario:** Caloric restriction (CR) and time-restricted eating (TRE) strategies are equally effective in improving body weight, body composition, liver fat, and metabolic parameters. This outcome would suggest that the benefits of TRE are mainly driven by an inadvertent reduction in energy intake that typically accompanies a shorter eating window.

• **Superiority of the CR group**: A 25% caloric restriction results in significantly greater improvements in the primary outcomes compared with TRE. This would indicate that the energy deficit is the fundamental driver of metabolic health and that chronobiological alignment alone does not confer significant additional benefits over traditional caloric reduction.

• **Superiority of the TRE group**: Alignment with circadian rhythms and a 14-hour fasting period confers metabolic and molecular advantages that surpass the effects of a standard caloric deficit. In this case, the TRE group would demonstrate a greater capacity to slow biological aging (BoA), preserve lean mass, and reduce liver fat, even if total weight loss were similar to that of the CR group, thereby validating the geroscience hypothesis regarding the importance of nutritional chronobiology.

## Analytical approach

### Intention to treat analyses

The primary analysis will be conducted according to the **intention-to-treat (ITT)** principle. This means that all randomized participants will be included in their originally assigned groups, regardless of their level of adherence or whether they completed the study. To assess the effects of the intervention on primary and secondary outcomes, linear mixed-effects models will be used, incorporating the interaction between intervention arm and time as fixed effects and a subject-level random intercept to model repeated measures. Missing data will be managed using a random forest–based imputation technique, which is robust for managing different data types and non-linear relationships.

### Per protocol analyses

**Per-protocol** analyses will be conducted, excluding non-adherent participants, to verify the robustness of the results. This approach will allow assessment of the efficacy of the intervention among subjects who strictly complied with the assigned protocol. To apply the per-protocol analysis, operational adherence thresholds will be defined to identify participants considered adherent in each intervention arm, in order to evaluate intervention efficacy under optimal compliance conditions:

• **Caloric restriction group (CR / CRG):** A participant will be considered adherent to the protocol when they achieve and maintain a reduction in energy intake of ≥ 20% relative to baseline energy intake, as assessed from dietary records and/or established monitoring tools, for at least 80% of the intervention period.

• **Time-restricted eating group (TRE):** Adherence will be defined as maintaining a daily fasting window of ≥ 13 hours (equivalent to an eating window of ≤ 11 hours) for at least 80% of the monitored days throughout the intervention, regardless of changes in total energy intake.

These thresholds will allow identification of subjects who strictly complied with the assigned strategy and, in the case of the TRE group, disentangle the effect of the temporal eating pattern from potential spontaneous reductions in total energy intake, thereby strengthening the causal interpretation of the results in the per-protocol analyses.

### As per treatment analyses

An **as-treated** analysis will also be conducted, classifying participants according to their adherence to the Mediterranean diet, caloric intake, and eating window, independently of the originally assigned intervention.

Accordingly, all subjects who reduced their energy intake by ≥20% and maintained an eating window of ≥12 hours per day will be assigned to the CR group. All subjects who extended their fasting window to ≥13 hours per day and reduced caloric intake by ≤10% will be assigned to the TRE group. An additional group will be created including participants who extended their fasting window to ≥13 hours per day and reduced their energy intake by ≥20% (CR+TRE group). Participants who maintained an eating window of ≥12 hours per day and reduced caloric intake by ≤10% will be included in the comparison group.

### Stratified analyses

In addition, **stratified analyses by sex** will be conducted. Beyond sex-stratified analyses, in a nutritional intervention study such as the one described, it is methodologically highly relevant to explore subgroup analyses based on clinical, metabolic, and lifestyle variables that may modulate the response to the intervention or function as effect modifiers. The following particularly relevant and justified stratification variables are proposed:

• **Baseline clinical conditions:** These allow evaluation of whether intervention efficacy differs according to prior health status.

- Hypertension (presence/absence)

- Type 2 diabetes (presence/absence)

- Dyslipidaemia (presence/absence; hypertriglyceridemia, elevated LDL)

- Metabolic syndrome (according to ATP III or IDF criteria (11))

- Hepatic steatosis (presence/absence, or by grade if applicable)

• **Body weight status and body composition:** As the effects of strategies such as caloric restriction or TRE may depend on baseline adiposity:

- BMI categories: Overweight (25–30 kg/m²), obesity (by grade)

- High vs. normal visceral fat

• **Baseline metabolic profile**:

- Fasting glucose or HbA1c (clinical cut-off points)

- Baseline lipid profile (high triglycerides, LDL and HDL by clinical cut-offs)

• **Age (beyond randomization stratification):** Although used for randomization, it may be informative to analyse 50–59 vs. 60–70 years, assessing potential differences in metabolic adaptations, adherence, or magnitude of effect.

• **Baseline physical activity level** (median and tertiles of METs/min/week) and sedentary behaviour (<7 h vs. ≥7 h of sedentary time per day)

• **Chronotype or sleep habits** (if available): Particularly relevant for the TRE group:

- Morning vs. evening chronotype according to the classification defined by the CEE questionnaire

- Sleep duration (<8 h vs. ≥8 h per day) or sleep quality according to the Pittsburgh questionnaire score and objective accelerometry data

These analyses are conceived as secondary or exploratory analyses, specified a priori. The number of subgroups will be limited to ensure adequate statistical power.

### Adjustment strategies

To ensure the robustness of the findings and to accurately isolate the effect of the nutritional interventions (TRE and CR) on aging biomarkers and metabolic health, the ENSATI trial analysis plan will implement a hierarchical adjustment strategy composed of five progressive models. Each successive model incorporates the variables from the previous one, allowing evaluation of how the inclusion of different confounding factors influences effect estimates:

• **Model 1 (Unadjusted Analysis):** This level represents the basic analysis without additional adjustments, following the intention-to-treat principle to observe the direct association between the assigned intervention arm and the primary or secondary outcomes.

• **Model 2 (Minimal Adjustment):** Adjusted for fundamental biological and structural variables: age and sex. In addition, this model incorporates adjustment for cluster membership (to account for correlation among members of the same household randomized together) and for the recruiting primary care centre (as a random effect).

• **Model 3 (Sociodemographic Adjustment):** Adds variables reflecting the participant’s social context to the previous model, with particular emphasis on educational level. This factor is crucial to control for potential biases related to access to nutritional information or socioeconomic disparities that may affect baseline health.

• **Model 4 (Lifestyle Adjustment):** Includes, in addition to the above, smoking status (current smoker, former smoker, or never smoker). This adjustment is essential given the direct impact of tobacco use on systemic inflammation and biological aging.

• **Model 5 (Maximum Adjustment Model):** This is the level of greatest analytical complexity. In addition to all previous variables, it adjusts for:

- Baseline clinical variables: Initial body weight, body mass index (BMI), and the presence of baseline comorbidities.

- Baseline dietary variables: Baseline adherence to the Mediterranean diet and initial total energy intake.

- Physical activity variables: Adjustment for both baseline physical activity and longitudinal changes in physical activity levels recorded during the 6-month intervention.

This sequential approach, evaluated using linear mixed-effects models, ensures that observed benefits in weight, liver fat, or epigenetic clocks are attributable specifically to the nutritional intervention and not to residual differences in behaviour or baseline participant characteristics. To avoid overfitting in the more complex models, regularized regression techniques and model selection based on information criteria such as AIC and BIC will be applied. To control the false discovery rate arising from multiple testing, the Benjamini–Hochberg procedure will be used.

As a complement to sensitivity analyses and to assess the robustness of associations to potential bias from unmeasured variables, the inclusion of the E-value is proposed. The E-value represents the minimum strength of association that an unmeasured confounder would need to have with both the intervention and the outcome (e.g., improvement in liver fat or reduction in biological age) to fully explain the observed association, conditional on the variables already adjusted for in the models.

• **Calculation description**: For a point estimate expressed as a Relative Risk (RR), the E-value is calculated as:

E = RR + RR × (RR − 1).

If the effect estimate is protective (RR < 1), the calculation is performed using the inverse value (1/RR).

• **Interpretation**: The higher the E‑value, the more robust the association, as an extremely strong confounder would be required to invalidate the result.

This analysis allows the results to be independent of the presence of confounding variables that could not be collected in the eCRFs or included in the maximum adjustment model (Model 5), which already accounts for factors such as age, sex, education, smoking, diet, weight, and physical activity. The inclusion of this metric strengthens the scientific validity of the trial by quantifying how likely it is that an unknown external factor could alter conclusions regarding the effectiveness of time-restricted eating (TRE) or caloric restriction (CR).

To prevent **overfitting** in the study analyses, particularly when using models with multiple adjustment variables, the following technical strategies will be implemented:

• **Regularized regression:** Regularization techniques will be applied to penalize excessive model complexity, ensuring stable and generalizable estimates.

• **k-fold cross-validation:** This procedure will be used as a complementary method to evaluate model performance across different subsets of the data, ensuring that results do not depend on a specific sample partition.

• **Model selection criteria**: Final model selection will be based on robust statistical information criteria, specifically the Akaike Information Criterion (AIC), the Bayesian Information Criterion (BIC), and the conditional AIC.

These methodologies make it possible to balance model goodness of fit with simplicity, avoiding models that “learn” data noise rather than the true biological associations between nutritional interventions and aging biomarkers.

## Temporal framework of the analyses

The temporal framework for analyses and data management in the ENSATI trial has been designed to ensure data integrity and timely responsiveness to incidents over the 12-month duration of the study.

• **Monthly Monitoring and Data Cleaning (Months 1–6)**

During the active intervention phase, data are collected using electronic case report forms (eCRFs). On a monthly basis, these data are downloaded to perform a partial quality-oriented analysis. This process allows for early identification of missing data, implausible values, deviations in adherence, and adverse effects. The personnel responsible for this monthly review are not blinded to group assignment. This team prepares a technical report that is shared with the nutritionists and dietitians involved in fieldwork. The report is subsequently reviewed together with the principal investigator to implement the following control actions:

- Review inconsistent data directly in the original records.

- Contact participants to retrieve missing information.

- Design and implement rescue strategies to improve protocol adherence.

• **Transition and Database Lock (Months 6–12)**

Once the last patient completes the 6-month intervention phase, the frequency of these reviews is reduced to a quarterly basis. At the end of the 12-month follow-up period for the last participant and once all data entry in the eCRFs has been completed, a final data download is performed. At this stage, definitive data cleaning and curation are conducted, including correction of residual errors and entry of any remaining missing data to consolidate the database.

• **Blinding and Statistical Analysis**

After completion of data curation, the study is blinded. To ensure impartiality, a random sequence is generated to assign the letters A, B, and C arbitrarily to each intervention arm. The resulting fully blinded database is then delivered to the statistical analysis team, who will perform the mixed-effects models and sensitivity analyses without knowledge of group identities.

• **Data processing timeline**

| Month | | | Phase | | | Main activities |  |
| --- | --- | --- | --- | --- | --- | --- | --- |
| 1 -6 | | Active intervention | | | • Data collection in the eCRF | | |
|  |  |  |  |  | • Monthly data download and quality | | |
|  |  |  |  |  | • Partial quality analysis (missing data, implausible values, adherence, adverse events) | | |
|  |  |  |  |  | • Monthly technical report and corrective actions | | |
|  |  |  |  |  | • Contact with participants if needed | | |
|  |  |  |  |  | • Adherence rescue strategies | | |
| 6 | Active intervention / Transition | | | • Final intensive monthly quality control | | | |
|  |  |  |  | • Progressive closure of the intervention phase (for participants completing 6 months) | | | |
| 7 - 12 | Follow-up | | | • Quarterly database review | | | |
|  |  |  |  | • Consistency checks and resolution of pending queries | | | |
|  |  |  |  | • Entry of follow-up data | | | |
|  |  |  |  | • Resolution of detected issues | | | |
|  |  |  |  | • Assessment of data completeness and quality | | | |
|  |  |  |  | • Consolidation of final follow-up data | | | |
|  |  |  |  | • Preparation for definitive database closure | | | |
|  |  |  |  | • Final verification of internal data consistency | | | |
| 12 | Closure and analysis | | | • Completion of follow-up for the last participant | | | |
|  |  |  |  | • Final database download | | | |
|  |  |  |  | • Definitive data cleaning and curation | | | |
|  |  |  |  | • Database lock | | | |
|  |  |  |  | • Blinding (A/B/C) | | | |
|  |  |  |  | • Handover to the statistical team and analyses | | | |

eCRF: electronic case report form

# **Statistical principles**

## Treatment of variables

The statistical treatment of the variables will follow a systematic approach aimed at ensuring the validity of descriptive and comparative analyses, as well as the appropriate interpretation of results from the outset of the study. First, all continuous variables will undergo an initial exploration to assess their distribution using graphical methods (histograms, Q–Q plots) and formal normality tests, such as the Shapiro–Wilk test. This evaluation will allow variables to be classified according to whether or not they conform to a normal distribution.

Continuous variables that show an approximately normal distribution will be described using means and standard deviations, whereas those that do not meet normality assumptions will be summarized using medians and interquartile ranges. In cases where a non-normally distributed continuous variable is relevant for inferential analyses, the application of mathematical transformations (e.g., logarithmic, square root, or Box–Cox transformations) will be considered to approximate normality and enable the use of parametric models when methodologically appropriate. If, after transformation, the variable still does not conform to a normal distribution, non-parametric methods will be applied.

Categorical variables will be described using absolute counts and percentages.

## Descriptive analyses of the population

The first descriptive table of the study will comprehensively present sociodemographic, clinical, medication-related, and lifestyle variables, including, among others, baseline adherence to the Mediterranean diet, smoking status, sleep patterns, and physical activity level. In addition, this table will include the number of participants assigned to each intervention arm, as well as the number of participants randomized individually or as part of a family unit (cluster).

For baseline comparisons of these variables across the different intervention arms, statistical tests appropriate to the nature and distribution of the data will be used. For continuous variables with a normal distribution, parametric tests such as analysis of variance (ANOVA) will be applied. For non-normally distributed continuous variables, equivalent non-parametric tests, such as the Kruskal–Wallis test, will be used. Categorical variables will be compared using the chi-square test or, when expected cell frequencies are low, Fisher’s exact test. This approach will allow assessment of baseline balance between groups and exclusion of systematic differences prior to the intervention.

## Missing data treatment

Regarding the handling of missing data, an imputation method based on random forest algorithms will be used, which is particularly well suited for datasets containing mixed variables (continuous and categorical) and potentially non-linear relationships among them. This approach allows preservation of the multivariate structure of the data and minimizes bias associated with missing information, thereby contributing to the robustness of subsequent analyses.

- **Assumptions and Scope of Imputation**: The handling of missing data in the ENSATI trial will operate under the Missing At Random (MAR) assumption, where the probability of a value being missing is related to the observed data rather than the missing values themselves.

- V**ariables included in the imputation model**: To maintain the integrity of the causal inference and avoid the artificial inflation of treatment effects, imputation will be strictly limited to non-dependent variables, specifically the confusing (confounding) variables required for the hierarchical adjustment models. Primary and secondary outcome measures (dependent variables) will not be imputed in the main analysis to prevent the introduction of synthetic longitudinal trajectories. The random forest model will utilize the multivariate structure of the dataset to estimate missing values for the following confounding factors:

- Sociodemographic Factors: Educational level, years of schooling, employment status, and household size.
- Lifestyle and Behavioral Factors: Baseline smoking status and baseline physical activity levels (METs/min/week).
- Baseline Clinical and Dietary Factors: Baseline weight, BMI, comorbidities (hypertension, diabetes), and initial Mediterranean diet adherence (MEDAS) or total energy intake.

- **Methodological Rationale and Handling of Uncertainty:** A random forest–based imputation method (such as the missForest algorithm) will be employed because it is non-parametric and uniquely suited for the high-dimensional, mixed-type data (continuous clinical measures and categorical molecular profiles) inherent in geroscience. Unlike linear imputation methods, this approach:

- Captures Non-linear Relationships: Effectively models complex interactions between lifestyle confounders and biological aging signatures without pre-specifying a functional form.
- Preserves Multivariate Structure: Uses the interdependencies across all observed variables to provide a more accurate estimation of missing entries.
- Addresses Uncertainty: Uncertainty is managed through the iterative nature of the forest construction, which reduces prediction error by averaging across a large ensemble of decision trees, thereby minimizing bias associated with missing information.

- **Validation through Sensitivity Analyses**: To quantify the impact of the imputation strategy and assess the robustness of the results, the following conventional approaches will be used alongside the imputed Intention-to-Treat (ITT) model, Completers-Only Analysis and Per-Protocol Analysis.

Additionally, E-value will be calculated to determine the strength an unmeasured or poorly imputed confounder would need to have to negate the observed treatment effect, providing an additional layer of evidence for the study’s internal validity.

## Confidence intervals and p value

### Primary outcomes

Given that the list of primary outcomes—which includes changes from baseline to 6 months in body weight, body composition (fat mass and lean mass), liver fat, and basal metabolism—has been defined a priori, and that there is substantial evidence in the literature suggesting a potential beneficial effect of nutritional interventions on these parameters, **no adjustment for multiplicity will be required** for this set of variables. Results will be presented as point estimates accompanied by their corresponding 95% confidence intervals. p‑values lower than 0.05 will be considered statistically significant.

### Secondary outputs

For secondary outcomes, which encompass dimensions such as cardiometabolic markers, dynamic glycaemic control, faecal microbiota, molecular biomarkers of aging, and cognitive health, two types of confidence intervals will be reported: nominal confidence intervals and, exclusively as auxiliary analyses, confidence intervals adjusted for multiple testing. The nominal 95% confidence intervals will describe the results of the assessment of each individual outcome. In addition, multiplicity-adjusted confidence intervals based on the Bonferroni procedure (confidence intervals of 1−α/m, where m is the number of comparisons) will be estimated for the secondary outcomes, with m representing the total number of secondary variables analysed in the ENSATI protocol.

Reports and publications derived from the study will focus primarily on nominal confidence intervals, grounding the interpretation of findings in their coherence and biological plausibility; however, multiplicity-adjusted confidence intervals will be included for secondary outcomes solely as complementary or auxiliary analyses.

# **Methods to evaluate the effect of the intervention**

## Main model

For the evaluation of primary and secondary outcomes in the ENSATI trial—focused on longitudinal changes in biomarkers—**linear mixed-effects models with repeated measures** will be used to analyse the association between the intervention and the study variables. Follow-up time will be computed from the baseline visit (V_00) to the evaluation points at 6 and 12 months. The models will include a random intercept and will account for intra-cluster correlation among members of the same household, due to the family-based randomization strategy. Both within-group changes and differences between intervention arms (TRE, CRG, and CG) will be analysed, with the recruiting centre included as a random effect.

Regarding changes in body weight, a weight loss of 5% will be defined as clinically meaningful, based on robust evidence linking this threshold to substantial metabolic improvements and a reduced risk of conditions such as diabetes. As a complementary analysis, changes in BMI will be evaluated as a continuous variable using multilevel mixed-effects models, likewise considering a 5% reduction in BMI as a clinically relevant magnitude of change.

Finally, for changes in waist circumference and segmental body composition, a 5% reduction in waist circumference will be considered clinically significant, in line with results from landmark lifestyle interventions demonstrating protective benefits with reductions of similar magnitude. Likewise, for changes in **fat mass, a 5% reduction** **will be defined as a clinically relevant** threshold, following the same proportional logic applied to body weight and waist circumference in metabolically at-risk populations. With respect to **lean mass, maintenance will be defined as preservation of this tissue in the absence of a statistically significant loss compared with the active dietary counselling control group or baseline values**. The objective of the analysis plan is to confirm that the intervention strategies reduce adiposity without accelerating muscle mass loss relative to continuous energy restriction, thereby preserving functional capacity and prolonging participants’ healthy lifespan.

## Sensitivity analyses

As part of the **sensitivity analyses** to assess the **robustness** of the results, a “**completers-only**” approach will be employed. This method involves conducting the statistical analysis including only those participants who have complete data at the corresponding follow-up assessments, without applying imputation techniques for missing values. The aim of this approach is to verify whether the conclusions derived from the primary intention-to-treat (ITT) analysis—which uses random forest–based imputation for missing data—remain consistent when the sample is restricted to subjects who successfully completed the protocol. In this way, it is ensured that participant attrition during the study has not substantially biased the estimates of the intervention effects.

## Correlation matrix

Defining the correlation structure is an essential technical component for correctly analysing repeated-measures data, as observations taken from the same participant at different time points (baseline, 3, 6, and 12 months) are correlated with one another and are not independent. To address this interdependence, linear mixed-effects models will be used, allowing the relationship among repeated measures to be modelled through the following structures:

• **First-order autoregressive (AR(1)):** This structure is considered under the assumption that measurements taken closer in time (for example, between month 1 and month 2) are more strongly correlated than those separated by longer intervals (such as between baseline and month 12). It is an efficient model that assumes the correlation decreases in a predictable manner as temporal distance increases.

• **Unstructured covariance**: This is the most flexible and robust approach, as it does not impose any predefined pattern on the correlations among the different time points. It allows each pair of measurements to have its own covariance and unique variance. Although highly precise, it requires estimation of a larger number of parameters; therefore, its final selection will depend on information criteria such as AIC or BIC to avoid overfitting.

These structures are incorporated into models that include the interaction between intervention arm and time as fixed effects and a subject-level random intercept. The main objectives of specifying the correlation structure are:

- Modelling individual variability: To accurately capture the temporal trajectories of each participant.

- Statistical validity: To ensure that the resulting standard errors and p-values are correct, thus avoiding erroneous conclusions regarding the effectiveness of time-restricted eating (TRE) or caloric restriction (CR) in improving aging biomarkers and metabolic health.

- Data handling: To help ensure model robustness even in the presence of missing data, which will be addressed beforehand using random forest–based imputation.

## Mediation analyses

This analysis will be complemented by formal mediation analyses focused on the primary outcomes of the ENSATI study, including body weight, body composition, liver fat, and metabolism, as well as biomarkers of aging (BoA). The approaches proposed by Lange (2011) and Lin (2017) will be adapted, incorporating mediators and confounders that vary over time. These methods model mediation effects within a counterfactual framework, allowing estimation of the direct and indirect effects of the interventions (caloric restriction and time-restricted eating) on liver health and the deceleration of the biological clock. In this way, it will be possible to assess whether the metabolic and molecular benefits are achieved through weight loss or through improvements in diet quality and physical activity that operate beyond simple reductions in body weight.

# **Omics data analysis: metagenomics and epigenomics**

The analysis of omics data in the ENSATI trial, encompassing epigenomics and metagenomics, is designed to identify the molecular mechanisms underlying biological aging and its response to nutritional interventions. The specific methodology for these analyses and their longitudinal integration is detailed below:

## a. Epigenomic analyses (DNA Methylation)

The study focuses on the DNA methylation profile extracted from peripheral blood mononuclear cells (PBMCs) using the Infinium MethylationEPIC BeadChip platform.

• **Data processing**: This includes background correction, quantile normalization, and probe bias correction. Beta methylation values are calculated using the RCP method and converted into M values for statistical analysis.

• **Epigenetic clocks**: Biological ages will be estimated using first-, second-, and third-generation clocks, such as Horvath, GrimAge, PhenoAge, and DunedinPACE, in addition to inferring telomere length from methylation data.

• **Bioinformatic analysis**: Differentially methylated positions (DMPs) and regions (DMRs) will be identified, along with functional pathway enrichment analyses (GO, KEGG, Reactome).

## b. Faecal Metagenomics Analyses

To assess the diversity and function of the gut microbiome, faecal samples collected at the baseline visit and at 6 and 12 months will be analysed.

• **Sequencing**: Shotgun metagenomic sequencing will be performed using Illumina platforms, ensuring a minimum of 10 million reads per sample.

• **Diversity metrics**: Alpha and beta diversity indices will be calculated, along with taxonomic profiling and functional annotation of microbial genes.

• **Quality control**: Technical replicates, negative controls, and batch-effect correction procedures will be included.

## c. Integration with longitudinal analyses

The integration of these data with the main clinical outcomes is conducted within a temporal framework comprising 6 months of intervention and 6 months of follow-up.

• **Longitudinal mixed-effects models:** Changes in both DNA methylation and microbial abundance will be evaluated using linear mixed-effects models, incorporating the interaction between intervention arm and time as fixed effects and a subject-level random intercept.

• **Adjustment strategies:** These omics models will follow the same hierarchical adjustment structure as the clinical variables, reaching the maximum adjustment Model 5, which accounts for age, sex, education, smoking status, body weight, adherence to the Mediterranean diet, energy intake, and physical activity.

• **Multi-omics and artificial intelligence**: Multivariate methods and machine learning algorithms will be employed to explore mechanistic links between molecular patterns, adherence to the intervention (TRE or CR), and metabolic outcomes such as liver fat and body weight. Dimensionality reduction and variable selection techniques (such as LASSO/Elastic Net regularization and tree-based methods) will be applied, along with supervised models (random forest, gradient boosting) to predict key metabolic outcomes including liver fat, body weight, and other cardiometabolic parameters. These models will enable the capture of non-linear and highly complex relationships between omics and phenotypic variables. In addition, cross-validation strategies (k-fold cross-validation) will be implemented to evaluate predictive performance and model generalizability, minimizing overfitting. Unsupervised analyses (clustering) will also be explored to identify subgroups of individuals with homogeneous molecular and metabolic profiles, facilitating a more precise characterization of responders and non-responders to each intervention. Ultimately, these approaches will allow modelling of the underlying biological mechanisms linking adherence to dietary interventions, modulation of molecular patterns, and changes in clinical outcomes, thereby contributing to the development of precision nutrition strategies aimed at optimizing healthy aging.

• **Multiplicity control:** Due to the high volume of comparisons inherent to omics data, the Benjamini–Hochberg procedure will be applied to control the false discovery rate (FDR).

# **Graphical representation**

The graphical and tabular presentation of the ENSATI trial results has been designed to provide a clear and transparent overview of the effectiveness of the interventions (TRE and CR). In accordance with CONSORT guidelines, data presentation will be structured as follows:

## a. Tables

- **Baseline Characterization Table**: This will be the first table of the study and will compare sociodemographic variables (age, sex, education), clinical variables (body weight, BMI, liver fat), and lifestyle factors (diet, physical activity, smoking) across the three intervention arms.

- **Primary and Secondary Outcomes Table**: This table will present point estimates of changes at 6 and 12 months, accompanied by their 95% confidence intervals and p-values (or adjusted q-values) for each group.

- **Adherence and Diet Table**: A summary of actual energy intake, macronutrient composition, and compliance with the eating window.

- **Adverse Events Table**: This will display the counts and percentages of reported symptoms (fatigue, headaches, mood changes, etc.) by intervention group.

## b. Figures and graphical representations

To facilitate interpretation of the findings, different types of graphs will be used depending on the nature of the data:

• **Flow Diagram (CONSORT):** An essential figure detailing the number of participants assessed for eligibility, excluded, randomized, and those who completed follow-up at each study phase.

• **Trajectory Plots (Time-Series Line Graphs):** These will be used to display the temporal evolution of continuous variables such as body weight, fat mass, and basal metabolism. The plots will allow visualization of within-group changes and between-group differences at the 0, 3, 6, and 12-month time points.

• **Interaction Plots:** Specifically designed to represent the interaction between intervention arm and time, helping to identify whether the response to the dietary interventions varies significantly over the course of the study.

• **Forest Plots**: These will be used to summarize treatment effect estimates and their confidence intervals, facilitating visual comparison of intervention efficacy across different outcomes or subgroups (e.g., by sex or age).

• **Diagnostic Plots** (Residuals and Q–Q Plots): Used internally and in supplementary materials to demonstrate that the linear mixed-effects models meet assumptions of normality and homoscedasticity.

**• Multi-omics Visualizations:** For microbiota and epigenetic data, specific graphical representations will be used, such as heatmaps for differential abundance analyses and volcano plots to identify the most significant differentially methylated positions

# References

1. Drake JC, Yan Z. Targeting healthspan to optimally combat non-communicable disease in an aging world. *Sports Medicine and Health Science* (2019) 1:59–60. doi: 10.1016/j.smhs.2019.08.005

2. Mathur A, Taurin S, Alshammary S. New insights into methods to measure biological age: a literature review. *Frontiers in Aging* (2024) 5: doi: 10.3389/fragi.2024.1395649

3. Kraus WE, Bhapkar M, Huffman KM, Pieper CF, Krupa Das S, Redman LM, Villareal DT, Rochon J, Roberts SB, Ravussin E, et al. 2 years of calorie restriction and cardiometabolic risk (CALERIE): exploratory outcomes of a multicentre, phase 2, randomised controlled trial. *Lancet Diabetes Endocrinol* (2019) 7:673–683. doi: 10.1016/S2213-8587(19)30151-2

4. Waziry R, Ryan CP, Corcoran DL, Huffman KM, Kobor MS, Kothari M, Graf GH, Kraus VB, Kraus WE, Lin DTS, et al. Effect of long-term caloric restriction on DNA methylation measures of biological aging in healthy adults from the CALERIE trial. *Nat Aging* (2023) doi: 10.1038/s43587-022-00357-y

5. Heilbronn LK, Smith SR, Martin CK, Anton SD, Ravussin E. Alternate-day fasting in nonobese subjects: effects on body weight, body composition, and energy metabolism1,2. *Am J Clin Nutr* (2005) 81:69–73. doi: 10.1093/ajcn/81.1.69

6. Wilkinson MJ, Manoogian ENC, Zadourian A, Lo H, Fakhouri S, Shoghi A, Wang X, Fleischer JG, Navlakha S, Panda S, et al. Ten-Hour Time-Restricted Eating Reduces Weight, Blood Pressure, and Atherogenic Lipids in Patients with Metabolic Syndrome. *Cell Metab* (2020) 31:92-104.e5. doi: 10.1016/j.cmet.2019.11.004

7. Lowe DA, Wu N, Rohdin-Bibby L, Moore AH, Kelly N, Liu YE, Philip E, Vittinghoff E, Heymsfield SB, Olgin JE, et al. Effects of Time-Restricted Eating on Weight Loss and Other Metabolic Parameters in Women and Men With Overweight and Obesity: The TREAT Randomized Clinical Trial. *JAMA Intern Med* (2020) 180:1491–1499. doi: 10.1001/jamainternmed.2020.4153

8. Dote-Montero M, Clavero-Jimeno A, Merchán-Ramírez E, Oses M, Echarte J, Camacho-Cardenosa A, Concepción M, Amaro-Gahete FJ, Alcántara JMA, López-Vázquez A, et al. Effects of early, late and self-selected time-restricted eating on visceral adipose tissue and cardiometabolic health in participants with overweight or obesity: a randomized controlled trial. *Nat Med* (2025) 31:524–533. doi: 10.1038/s41591-024-03375-y
