# Supplementary figures and images for: Time-restricted eating versus calorie restriction for improving biomarkers of age in adults with overweight or obesity and incipient fatty liver disease: protocol for the ENSATI randomized controlled parallel groups trial
Source: Front Endocrinol (Lausanne). 2026 Jun 12;17:1849550. doi: 10.3389/fendo.2026.1849550 (PMC13303218; doi:10.3389/fendo.2026.1849550)

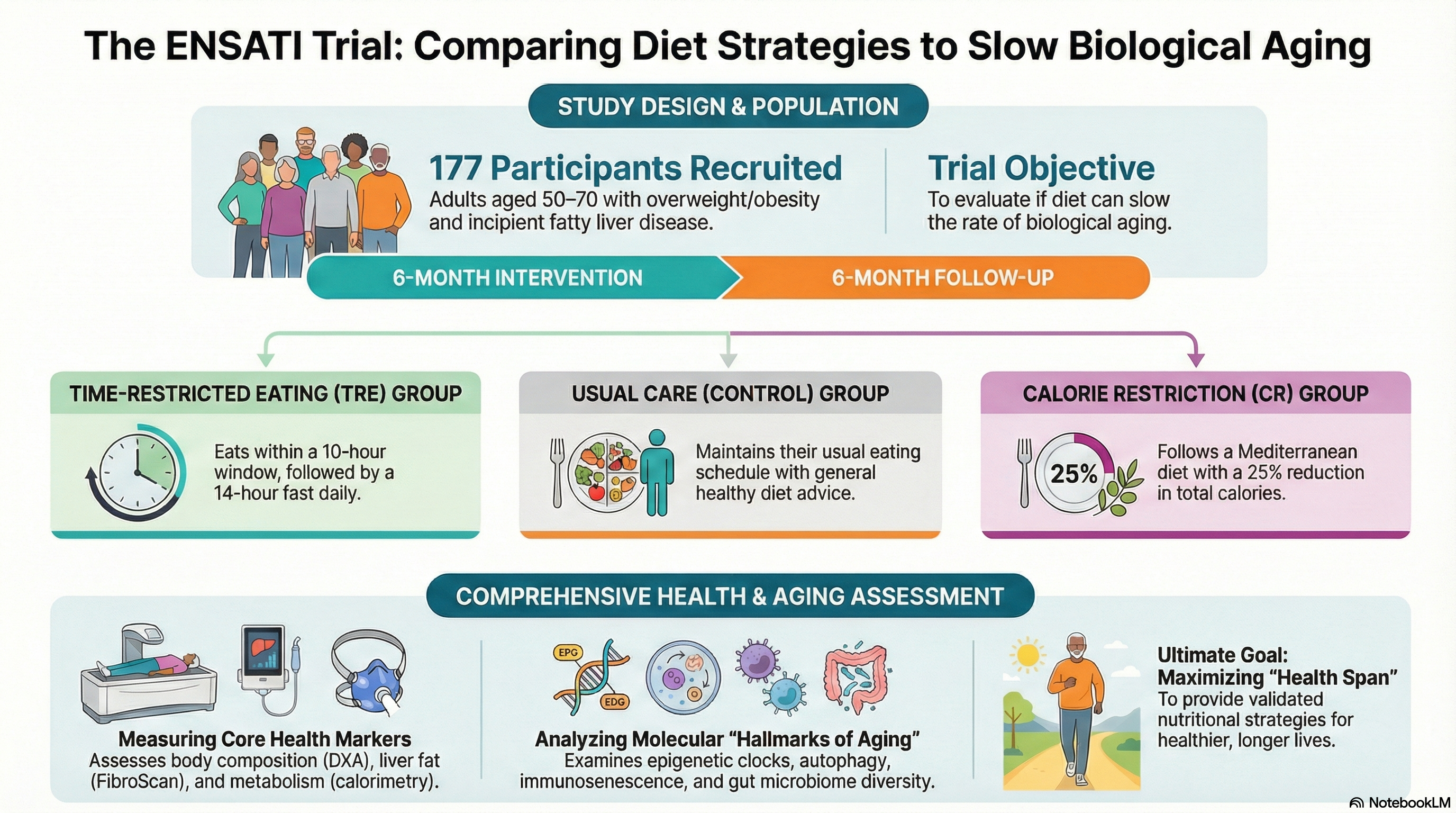

Supplement: Supplementary file 2 [file Image1.png]
